# AUP1 and UBE2G2 complex targets STING signaling and regulates virus-induced innate immunity

**DOI:** 10.1128/mbio.00602-25

**Published:** 2025-04-16

**Authors:** Xin-Tao Wang, Xi Zhu, Zhong-Hao Lian, Qian Liu, Hui-Hui Yan, Ye Qiu, Xing-Yi Ge

**Affiliations:** 1 Hunan Provincial Key Laboratory of Medical Virology, Institute of Pathogen Biology and Immunology, College of Biology, Hunan University 12569, Changsha, Hunan, China; Griffith University-Gold Coast Campus, Gold Coast, Queensland, Australia

**Keywords:** AUP1, UBE2G2, STING, innate immunity, virus infection

## Abstract

Stimulator of interferon genes (STING) is an endoplasmic reticulum (ER) signaling adaptor that is essential for the host immune response triggered by DNA pathogens. Precise regulation of STING is crucial for maintaining a balanced immune response and preventing harmful autoinflammation. Activation of STING requires its translocation from the ER to the Golgi apparatus. However, the mechanisms that maintain STING in its resting state remain largely unclear. Here, we find that deficiency of the ancient ubiquitous protein 1 (AUP1) causes spontaneous activation of STING and enhances the expression of type I interferons (IFNs) under resting conditions. Furthermore, deficiency of *UBE2G2*, a cofactor of AUP1, also promotes the abnormal activation of STING. *AUP1* deficiency significantly enhances STING signaling induced by DNA virus, and *AUP1* deficiency exhibits increased resistance to DNA virus infection *in vitro* and *in vivo*. Mechanistically, AUP1 may form a complex with UBE2G2 to interact with STING, preventing its exit from the ER membrane. Notably, infection with the RNA virus vesicular stomatitis virus (VSV) promotes the accumulation of lipid droplets (LDs) and AUP1 proteins. Additionally, *AUP1* deficiency markedly inhibits the replication of VSV because AUP1 deficiency reduces lipid accumulation and alters the expression of lipid metabolism genes, such as carnitine palmitoyltransferase 1A (*CPT1A*), monoglyceride lipase (*MGLL*), and sterol regulatory element-binding transcription factor 1 (*SREBF1*). This study uncovers the essential roles of AUP1 in the STING signaling pathway and lipid metabolism pathway, highlighting its dual role in regulating virus replication.

**IMPORTANCE:**

The stimulator of interferon genes (STING) signaling cascade plays an essential role in coordinating innate immunity against DNA pathogens and autoimmunity. Precise regulation of the innate immune response is essential for maintaining homeostasis. In this study, we demonstrate that ancient ubiquitous protein 1 (AUP1) and UBE2G2 act as negative regulators of the innate immune response by targeting STING. Notably, AUP1 interacts with STING to retain STING in the endoplasmic reticulum (ER), preventing STING translocation and thereby limiting STING signaling in the resting state. In addition, deficiency of *AUP1* markedly inhibits the replication of DNA virus and RNA virus. Our findings provide new insights into the regulation of STING signaling and confirm AUP1 has a dual role in regulating virus replication.

## INTRODUCTION

Stimulator of interferon genes (STING), a key signaling adaptor protein in the cGAS-STING pathways, localizes to the endoplasmic reticulum (ER) membrane in its resting state (1). The STING signaling cascade plays a crucial role in coordinating innate immunity against pathogenic double-stranded DNA (dsDNA) and in regulating autoimmunity (2–4). Upon activation and subsequent exit from the ER, STING participates in two distinct cellular effect pathways. The first pathway involves the translocation of STING to the Golgi apparatus, leading to the induction of autophagy, which is an ancient antiviral function of STING. In contrast, the second pathway, initiated at the Golgi apparatus, promotes the transcriptional activation of innate immune genes (5, 6). Although these two pathways are independent, STING ultimately degrades in the lysosome (5, 7, 8). Pathogen-derived cytosolic dsDNA is recognized by cGAS, which catalyzes ATP and GTP to produce the second messenger, cyclic GMP-AMP (cGAMP). cGAMP then binds to STING on the ER, triggering its conformational change and activation (9, 10). Following STING activation, it exits from the ER and translocates to the trans-Golgi network (TGN), where STING recruits TANK-binding kinase 1 (TBK1) and interferon regulatory factor 3 (IRF3). Subsequently, STING is phosphorylated by TBK1 (11). Upon phosphorylation by TBK1, IRF3 homo-dimerizes and translocates into the nucleus, where it induces the expression of IFNs and pro-inflammatory cytokines (11). These cytokines subsequently promote the transcription of a wide range of antiviral and inflammatory genes, mediating innate antiviral immunity and inflammatory responses (12, 13).

In addition to playing a crucial role in protecting the host from DNA pathogens, STING is also involved in autoimmune diseases caused by self-DNA, such as Aicardi–Goutieres syndrome, systemic lupus erythematosus, and other type I interferonopathies (14, 15). Furthermore, mutations in STING have been identified in patients with STING-associated vasculopathy with onset in infancy (SAVI) and lupus-like symptoms (4, 16, 17). The activation of STING is a dynamic process, and its accurate regulation is essential for maintaining immune homeostasis. Recent studies have shown that STING interacts with the Ca^2+^ sensor stromal interaction molecule 1 (STIM1), and deficiency of STIM1 leads to spontaneous activation of STING and enhanced expression of type I interferons under resting conditions (18). The absence of Niemann-Pick type C1 (NPC1) causes STING to activate abnormally, independently of cGAS and cGAMP, inducing the production of interferon-stimulated genes (ISGs) in the resting state (19). Additionally, adaptor protein complex 1 (AP-1) regulates the termination of STING-dependent immune activation (20). In the resting state, Toll-interacting protein (TOLLIP) deficiency results in reduced STING protein levels in nonhematopoietic cells and tissues, rendering STING protein unstable in immune cells and leading to severely dampened STING signaling capacity (21). Thus, the precise regulation of STING activation is vital for maintaining immune homeostasis and ensuring an appropriate immune response. However, the mechanisms by which STING is maintained in the resting state remain largely unclear.

Ancient ubiquitous protein 1 (AUP1) localizes to both the ER and lipid droplets (LDs) (22, 23). Related research indicates that AUP1 physically associates with the mammalian HRD1-SEL1L complex, and its depletion impairs the degradation of misfolded ER proteins (23, 24). Recent studies have also shown that STING interacts with the HRD1-SEL1L complex, and deficiencies in HRD1 or SEL1L specifically enhance STING signaling and immunity against viral infection (25). Additionally, AUP1 has been reported to interact with autocrine motility factor receptor (AMFR), which catalyzes K27-linked polyubiquitination of STING, thereby influencing the innate immune antiviral response (26, 27). These findings suggest a potential relationship between AUP1 and STING. In fact, data from a recent study indicate that AUP1 may interact with STING and affect its trafficking (19). AUP1 contains a C-terminal domain that shares strong homology with a domain known as G2BR, which binds the E2 ubiquitin conjugases G2 (UBE2G2) (22). UBE2G2 serves as a cofactor for AUP1, and its protein stability is regulated by AUP1 (28). Moreover, recent mass spectrometry data suggest that human STING may interact with UBE2G2 (29). However, the role of the AUP1-UBE2G2 complex in STING biology remains unexplored.

Whether the AUP1-UBE2G2 complex regulates STING-dependent innate immune responses remains unclear. Here, we report that the loss of *AUP1* or *UBE2G2* enhances STING-mediated innate immune responses induced by HT-DNA, diABZI, or cGAMP. Furthermore, *AUP1* deficiency promotes the innate immune response triggered by DNA viruses. Mechanistically, the AUP1-UBE2G2 complex interacts with STING to restrict its exit from the ER, and the loss of *AUP1* or *UBE2G2* induces spontaneous activation of the STING signaling pathway in resting state. Consequently, we observed strong resistance to viral infections in *AUP1* knockout cells. These results suggest that AUP1 plays an important role in regulating innate immune responses.

## RESULTS

### Knockout of *AUP1* augments cGAS-STING signaling

**Fig 1:**
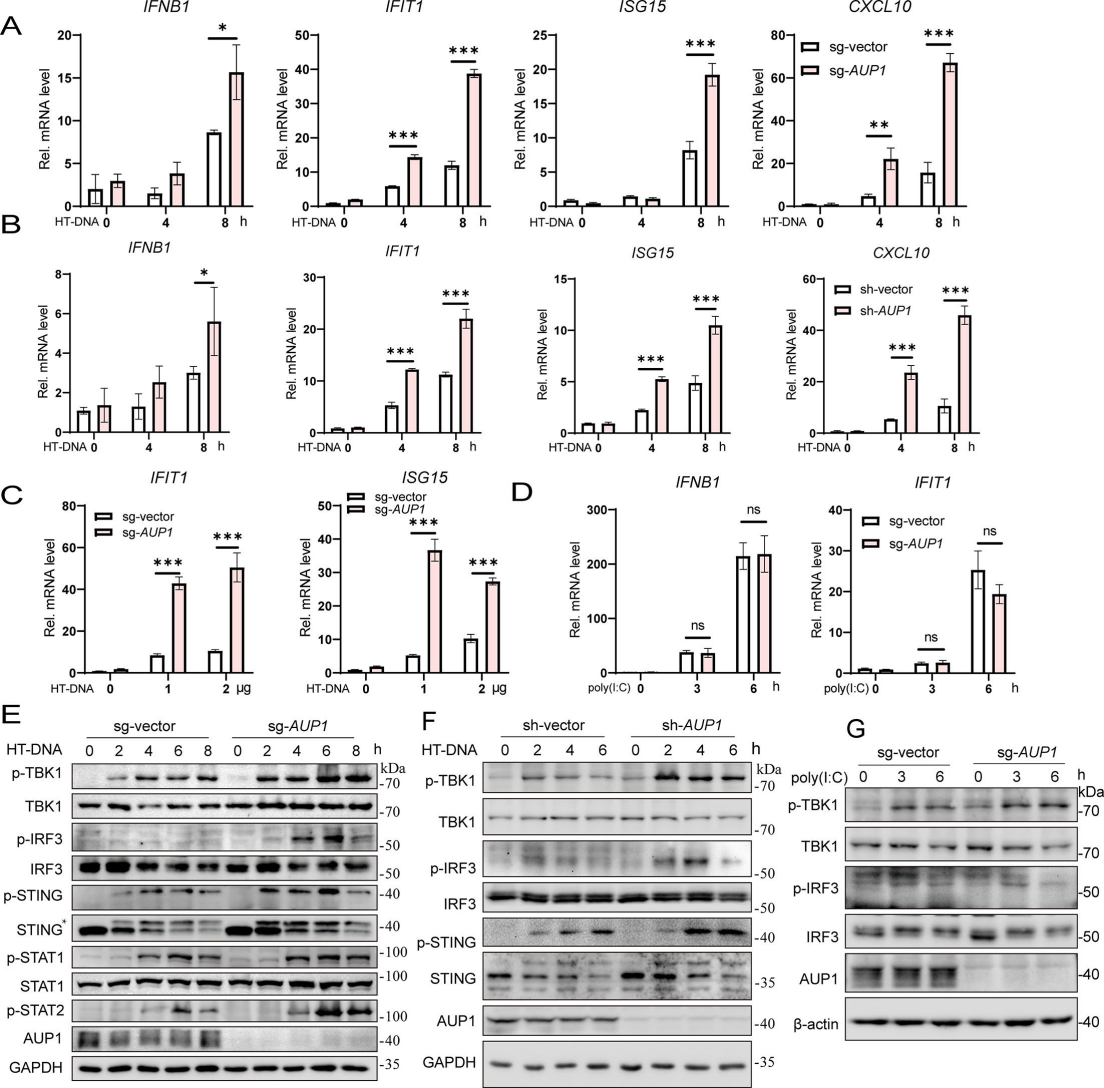
*AUP1* deficiency promotes the cGAS-STING signaling pathway. (**A**) Control cells or stable *AUP1*-deficient HeLa cells were transfected with HT-DNA (1 µg/mL) for indicated time points. The levels of *IFNB1*, *IFIT1*, *ISG15*, and *CXCL10* mRNA were examined by qRT-PCR. (**B**) Control cells or *AUP1*-knockdown HeLa cells were transfected with HT-DNA (1 µg/mL) for indicated time points. The levels of *IFNB1*, *IFIT1*, *ISG15*, and *CXCL10* mRNA were examined by qRT-PCR. (**C**) qRT-PCR analysis of *ISG15* and *IFIT1* mRNA abundance in *AUP1*-deficient HeLa cells and cells transfected with different doses of HT-DNA for 6 h. (**D**) qRT-PCR analysis of *IFNB1* and *IFIT1* mRNA abundance in wild-type and *AUP1*-deficient HeLa cells transfected with poly(I:C) for indicated time points. (**E**) Immunoblot analysis of the indicated proteins in wild-type and *AUP1*-deficient HeLa cells transfected with the HT-DNA for indicated time points. *, p-STING. (**F**) Immunoblot analysis of the indicated proteins in wild-type and *AUP*1 stable knockdown HeLa cells transfected with the HT-DNA for indicated time points. (**G**) Immunoblot analysis of the indicated proteins in wild-type and *AUP1*-deficient HeLa cells transfected with poly(I:C) for indicated time points. Data are representative of three experiments with similar results. Bar graphs show the means ± standard deviation (SD). *P < 0.05, **P < 0.01, and ***P < 0.001.

To investigate the role of AUP1 in innate immune signaling, we applied CRISPR-Cas9 gene editing technology to deplete *AUP1* expression (sg-*AUP1*), effectively knocking out AUP1 in HeLa cells (Fig. S1A). We observed that stimulation with the cGAS agonist herring testes DNA (HT-DNA) induced significantly higher expression of *IFNB1* and ISG mRNA levels in *AUP1* knockout HeLa cells compared to wild-type HeLa cells (Fig. 1A). To rule out the possibility of an off-target effect of *AUP1* sgRNA, we used shRNA to knock down the expression of *AUP1* in HeLa cells (Fig. S1B). Similar results were observed; knockdown of *AUP1* enhanced HT-DNA-stimulated *IFNB1* and ISGs mRNA levels compared to control HeLa cells (Fig. 1B). Furthermore, the expression of *IFIT1* and *ISG15* was significantly induced by different doses of HT-DNA in *AUP1* knockout HeLa cells compared to control cells (Fig. 1C). However, the expression levels of *IFNB1* and *IFIT1* in *AUP1* knockout cells treated with MDA5 and RIG-I agonist poly(I:C) were consistent with those of control cells showing no significant differences (Fig. 1D). Unsurprisingly, the phosphorylation of TBK1, IRF3, STING, and downstream proteins STAT1 and STAT2 in the JAK/STAT pathway was significantly enhanced after HT-DNA stimulation in *AUP1* knockout HeLa cells (Fig. 1E). Consistently, the phosphorylation of TBK1, IRF3, and STING was also significantly increased after HT-DNA stimulation in *AUP1* knockdown HeLa cells or HT1080 or L929 cells (Fig. 1F; Fig. S1C and D). Additionally, the knockout of *AUP1* had no marked effect on poly(I:C)-induced phosphorylation of TBK1 and IRF3 (Fig. 1G). Taken together, these data suggest that the knockout of *AUP1* specifically regulates the cGAS-STING signaling pathway but not the RLR signaling pathway.

### AUP1 interacts with STING

**Fig 2:**
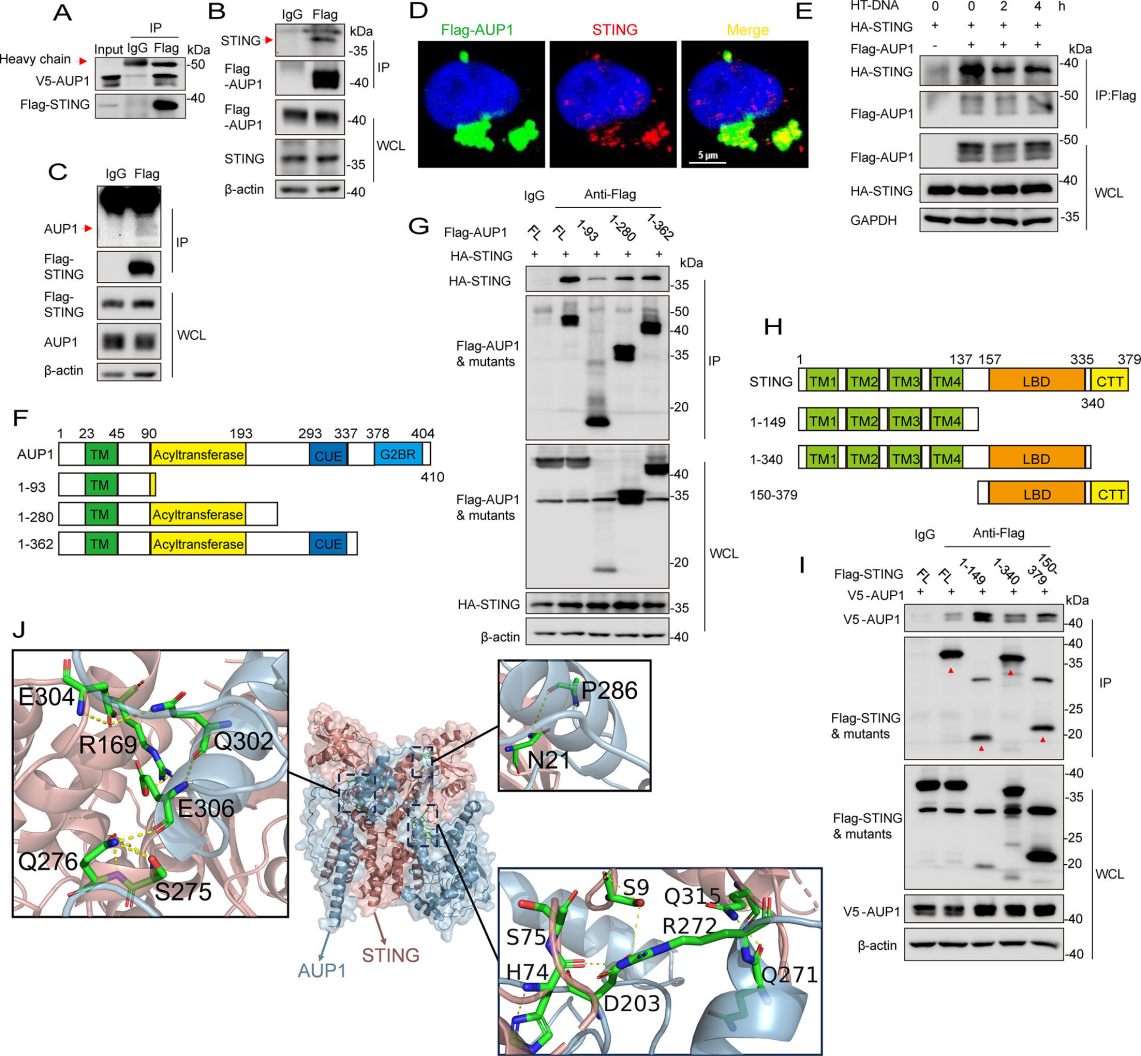
AUP1 interacts with STING. (**A**) HEK293T cells were transfected with the indicated plasmids. Twenty-four hours after transfection, the cell lysates were immunoprecipitated with an anti-Flag antibody or normal immunoglobulin G (IgG) and then immunoblotted with the indicated antibodies. (**B and C**) HeLa cells were transfected with the indicated plasmids. Twenty-four hours after transfection, the cell lysates were immunoprecipitated with an anti-Flag antibody or IgG and then immunoblotted with the indicated antibodies. (**D**) HeLa cells were stained with the indicated antibodies and imaged by confocal microscopy. Scale bar represents 5 µm. (**E**) HeLa cells were transfected with the indicated plasmids for 24 h and then transfected with HT-DNA for the indicated times, and the cell lysates were subjected to immunoprecipitation with the indicated antibodies. (**F**) Schematic of AUP1 and its truncation mutant protein domains. TM, transmembrane; CUE, coupling of ubiquitin conjugation to endoplasmic reticulum degradation; G2BR, UBE2G2 binding region. (**G**) Flag-tagged AUP1 or its mutants were individually transfected into HEK293T cells along with the HA-tagged STING. The cell lysates were immunoprecipitated with an anti-Flag antibody or IgG and then immunoblotted with the indicated antibodies. (**H**) Schematic of STING and its truncation mutant protein domains. TM, transmembrane; LBD, ligand-binding domain; CTT, carboxy-terminal tail. (**I**) Flag-tagged STING or its mutants were individually transfected into HEK293T cells along with V5-tagged AUP1. The cell lysates were immunoprecipitated with an anti-Flag antibody or IgG and then immunoblotted with the indicated antibodies. (**J**) 3D structures of AUP1/STING. Human STING crystal structure derived from PDB (6NT5). The structure of human AUP1 protein was derived from AlphaFold prediction.

Previous reports have suggested that AUP1 is a lipid droplet protein localized in the ER (22, 23). It is also well known that STING is localized in the ER. Given that both proteins share the same organelle localization, we hypothesized that there is an interaction between them. We next confirmed the interaction between AUP1 and STING using coimmunoprecipitation (co-IP) of ectopically or endogenously expressed proteins. The co-IP experiments demonstrated that exogenous Flag-STING interacts with exogenous V5-AUP1 in HEK293T cells (Fig. 2A). Interactions between exogenous Flag-AUP1 or Flag-STING and endogenous STING or AUP1 were confirmed in HeLa cells (Fig. 2B and C). Confocal microscopy revealed that STING co-localized with the Flag-AUP1 (Fig. 2D). Moreover, the interaction between AUP1 and STING was substantially decreased upon HT-DNA stimulation in HeLa cells (Fig. 2E). These experimental results indicate that there is indeed an interaction between AUP1 and STING, but this interaction weakens following STING activation.

To explore the interaction region between AUP1 and STING, we conducted a truncation experiment. Based on the functional region of AUP1 (22, 23, 30), we constructed three truncated plasmids, deleting the G2BR domain, deleting the G2BR and CUE domains, and retaining only the transmembrane domain (Fig. 2F). Interestingly, the truncation experiments showed that the full-length AUP1 and all its segments interact with STING, with the 1–93 domain of AUP1 exhibiting the weakest interaction (Fig. 2G). Next, we examined the interaction between different domains of STING and the full-length AUP1. Surprisingly, all truncated structures of STING interacted with AUP1, although the intensity of these interactions varied (Fig. 2H and I). A three-dimensional (3D) structural model of AUP1 and STING was generated, and docking simulation data revealed that the AAs Arg-272, Pro-286, Asp-203, Gln-271, Glu-306, and Gln-302 of AUP1, along with AAs His-74, Asn-211, Ser-75, Gln-315, Ser-275, Gln-276, Glu-304, Ser-9, and Arg-169 of STING, are responsible for the AUP1/STING interactions (Fig. 2J). These results indicate that AUP1 interacts with STING, involving multiple functional regions and interaction sites.

### Knockout of *AUP1* promotes STING signaling

To gain insights into the potential roles of AUP1 in innate immune responses, we examined the expression of various inflammatory cytokines in *AUP1*-deficient HeLa cells at the resting state. Among these, the transcripts of *IFNB1*, *IFIT1*, *ISG15*, and *CXCL10* were significantly increased in *AUP1*-deficient HeLa cells compared to wild-type cells (Fig. S2A). Additionally, the knockdown of *AUP1* resulted in increased transcription levels of *IFNB1* and *CXCL10*. Reintroducing wild-type AUP1 into *AUP1* knockdown cells led to a reduction in the expression of *IFNB1* and *CXCL10* (Fig. S2B).

**Fig 3:**
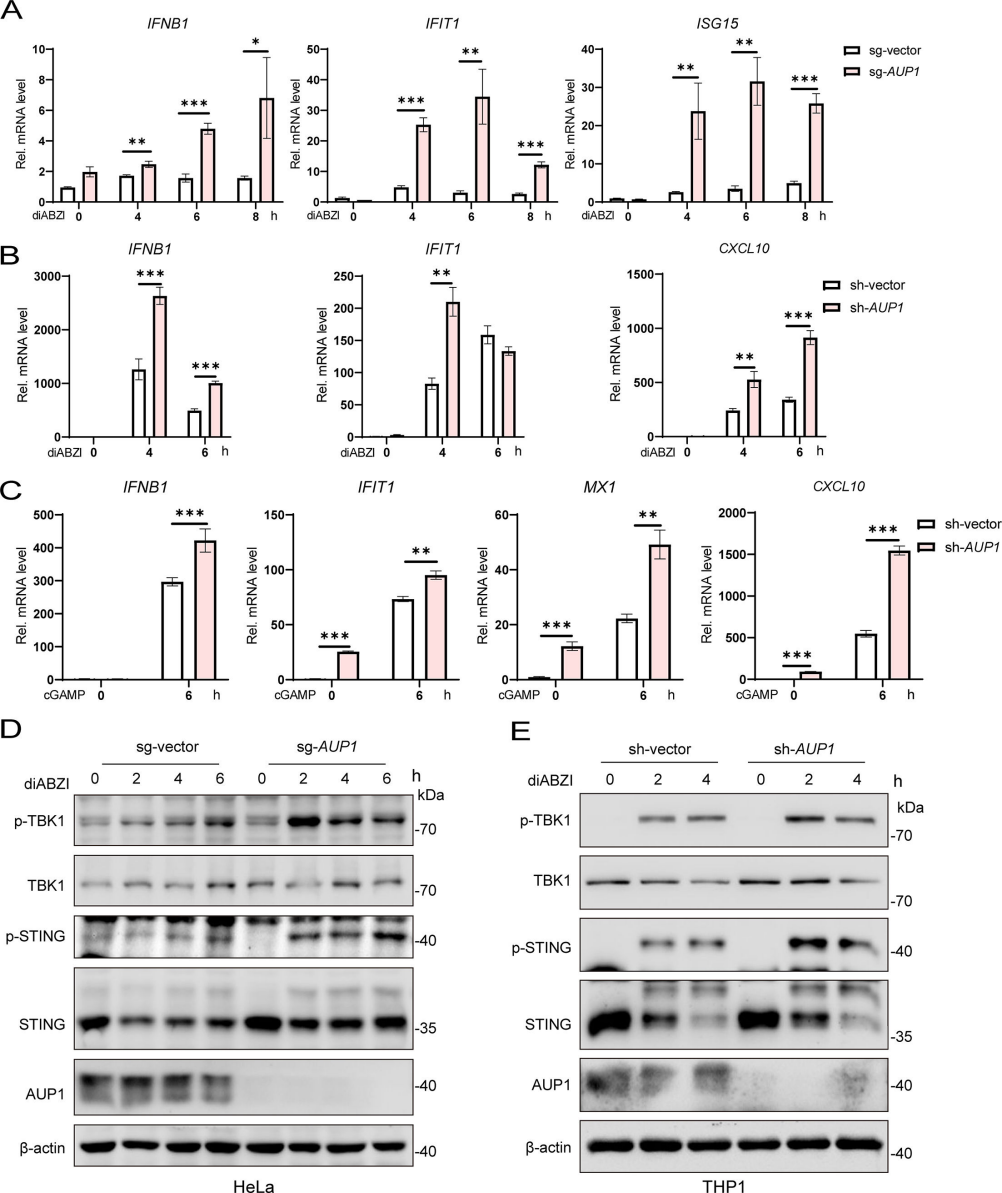
AUP1 restricts STING signaling. (**A**) qRT-PCR analysis of *IFNB1* and ISGs (*IFIT1* and *ISG15*) mRNA expression in wild-type and *AUP1* knockout HeLa cells after stimulation with diABZI (2.5 µM) for indicated time points. (**B and C**) qRT-PCR analysis of *IFNB1* and ISGs mRNA expression in wild-type and *AUP1* knockdown THP1 cells after stimulation with diABZI or 2′,3′-cGAMP (1 µg/mL) for indicated time points. (**D**) Immunoblot analysis of the indicated proteins in wild-type and *AUP1* knockout HeLa cells stimulated with diABZI for indicated time points. (**E**) Immunoblot analysis of the indicated proteins in wild-type and *AUP1* knockdown THP1 cells stimulated with diABZI for indicated times. Data are representative of three experiments with similar results. Bar graphs show the means ± SD. *P < 0.05, **P < 0.01, and ***P < 0.001.

To further confirm the effect of AUP1 on innate immunity regulated by STING, we treated the cells with STING agonist diABZI-C3 (hereafter, diABZI) (20), which is a small molecule agonist. Following diABZI stimulation, *IFNB1* and ISGs mRNA levels were significantly higher in *AUP1* knockout HeLa cell lines compared to the control cells (Fig. 3A). Next, we knocked down *AUP1* in THP1 (a human macrophage‐like cell line) cells. Similar results were observed. Following diABZI or cGAMP stimulation, *IFNB1* and ISGs mRNA levels were significantly higher in *AUP1* knockdown THP1 cell lines compared to the control cells (Fig. 3B and C). Additionally, the knockout of *AUP1* resulted in increased levels of p-TBK1 and p-STING and prolonged signaling compared to the stimulated control cells (Fig. 3D). Similar results were observed in *AUP1* knockdown THP1 cells, where the phosphorylation of TBK1 and STING was significantly enhanced after diABZI stimulation (Fig. 3E). These data suggest that AUP1 may modulate the innate immune response by acting on STING.

### UBE2G2 is required for AUP1 to regulate STING signaling

**Fig 4:**
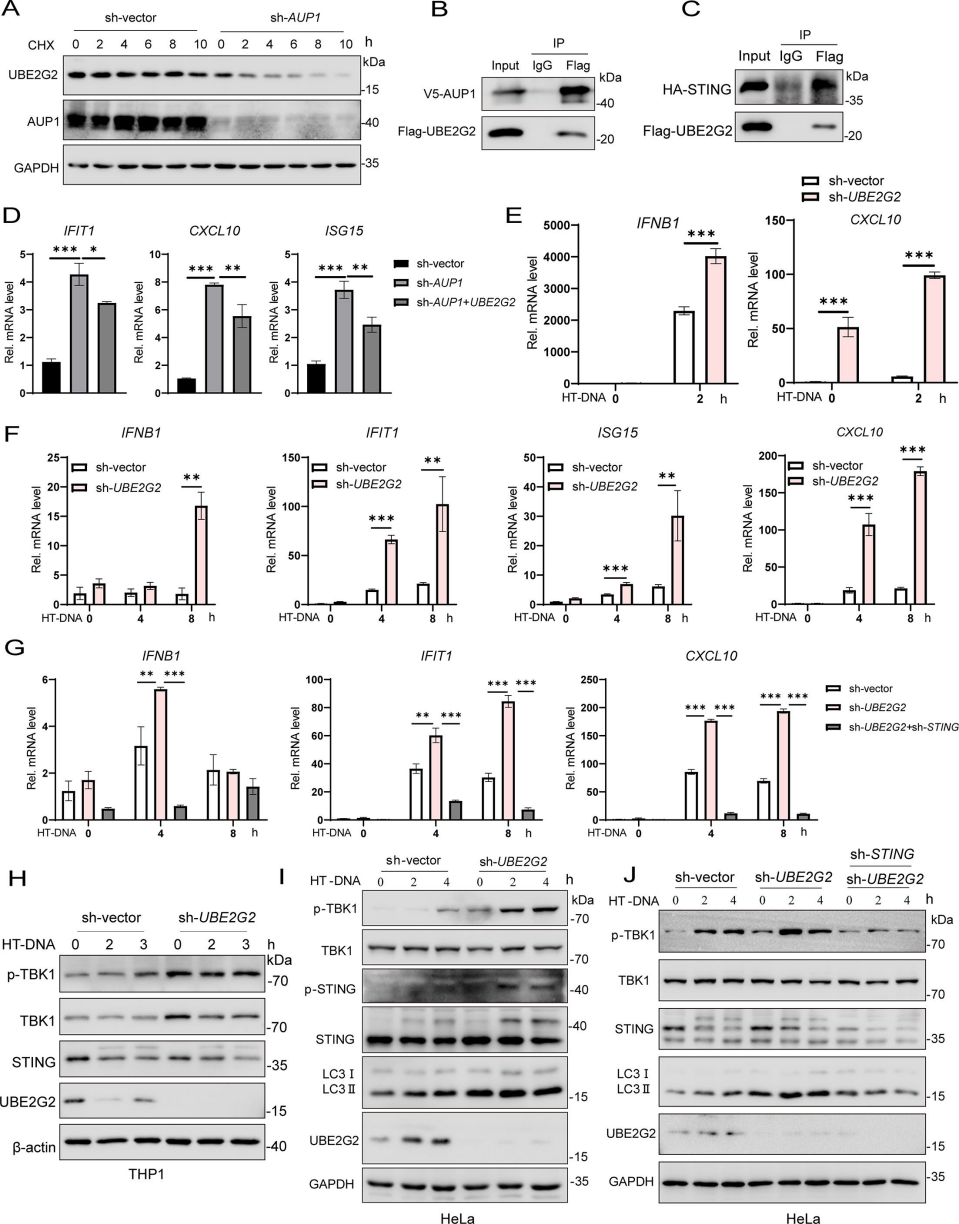
UBE2G2 is required for AUP1 to regulate STING signaling. (**A**) Cells were treated with CHX (50 µg/mL) for the indicated times to inhibit protein synthesis and UBE2G2 degradation monitored by Western blot in wild-type and *AUP1* knockdown HeLa cells. (**B and C**) HEK293T cells were transfected with the indicated plasmids. Twenty-four hours after transfection, the cell lysates were immunoprecipitated with an anti-Flag antibody or IgG and then immunoblotted with the indicated antibodies. (**D**) qRT-PCR analysis of the baseline *IFIT1, ISG15,* and *CXCL10* expression in *AUP1* knockdown cells, wild-type cells, and *AUP1* knockdown cells stably expressing wild-type *UBE2G2*. (**E**) qRT-PCR analysis of *IFNB1* and *CXCL10* mRNA expression in wild-type and *UBE2G2* knockdown THP1 cells after stimulation with HT-DNA for indicated times. (**F**) qRT-PCR analysis of *IFNB1* and ISGs (*IFIT1*, *ISG15,* and *CXCL10*) mRNA expression in wild-type and *UBE2G2* knockdown HeLa cells after stimulation with HT-DNA for indicated times. (**G**) qRT-PCR analysis of *IFNB1*, *IFIT1*, and *CXCL10* expression in wild-type, *UBE2G2* knockdown, and *UBE2G2* knockdown with knockdown of *STING* HeLa cells after stimulation with HT-DNA for indicated times. (**H and I**) Immunoblot analysis of the indicated proteins in wild-type and *UBE2G2* knockdown THP1 or HeLa cells after stimulation with HT-DNA for indicated times. (**J**) Immunoblot analysis of the indicated proteins in wild-type, *UBE2G2* knockdown, and *UBE2G2* knockdown with knockdown of *STING* HeLa cells after stimulation with HT-DNA for indicated times. Data are representative of three experiments with similar results. Bar graphs show the means ± SD. *P < 0.05, **P < 0.01, and ***P < 0.001.

As a cofactor of AUP1, UBE2G2 is closely related to AUP1, and AUP1 controls the protein stability of UBE2G2 (28). Therefore, we hypothesized that AUP1 may cooperate with UBE2G2 in regulating STING-mediated innate immune response. In a translation shut-off assay with CHX, UBE2G2 protein levels decreased in wild-type *AUP1* cells but became more unstable in *AUP1* knockdown cells, consistent with previously reported results (Fig. 4A). We also confirmed that UBE2G2 interacts not only with AUP1 but also with STING (Fig. 4B and C). Moreover, the knockdown of UBE2G2 did not affect the stability of AUP1 protein (Fig. S3A). *AUP1* deficiency significantly increased the expression of ISGs at resting state (Fig. S2A and B). However, reconstitution with wild-type UBE2G2 in *AUP1* knockdown HeLa cells reduced the expression of ISGs (Fig. 4D). Together, these data indicate that AUP1’s regulation of ISGs in the resting state may require the participation of UBE2G2.

We aimed to determine whether UBE2G2 regulates STING-mediated innate immune response. To do this, we transfected HT-DNA to activate the cGAS-STING pathway and observed that HT-DNA stimulation consistently triggered significantly higher expression levels of *INFB1* and *CXCL10* mRNAs in *UBE2G2* knockdown THP1 cells compared to wild-type *UBE2G2* cells (Fig. 4E). Consistently, the knockdown of *UBE2G2* enhanced HT-DNA-stimulated *IFNB1* and ISGs mRNA levels compared to control HeLa cells (Fig. 4F). Further knockdown of *STING* significantly reduced HT-DNA-induced expression of *IFNB1* and ISGs mRNA in *UBE2G2* knockdown cells (Fig. 4G). Consistently, the phosphorylation of TBK1 induced by HT-DNA stimulation was markedly enhanced in *UBE2G2* knockdown cells; however, the knockdown of *STING* inhibited HT-DNA-induced phosphorylation of TBK1 in these cells (Fig. 4H through J). These results suggest that knockdown of *UBE2G2* enhances the innate immune response, which is dependent on STING.

### Knockdown of *UBE2G2* activates STING signaling and autophagy

**Fig 5:**
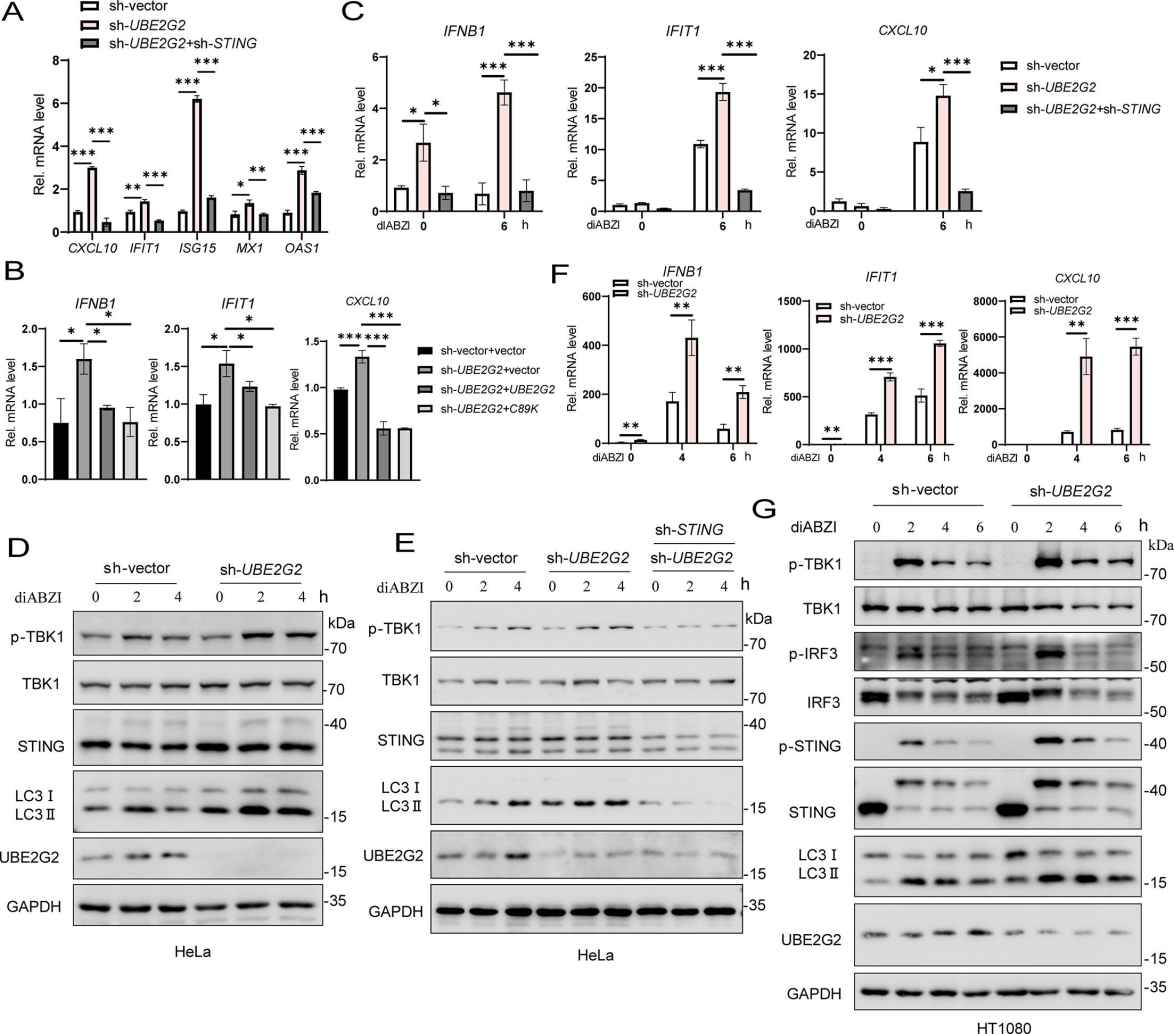
Knockdown of *UBE2G2* activates STING signaling. (**A**) qRT-PCR analysis of the baseline ISGs (*CXCL10*, *IFIT1*, *ISG15*, *MX1*, and *OAS1*) expression in wild-type cells and *UBE2G2* knockdown cells. (**B**) qRT-PCR analysis of the baseline *IFNB1* and ISGs (*IFIT1*, *CXCL10*) expression in wild-type cells, *UBE2G2* knockdown cells, and *UBE2G2* knockdown cells stably expressing wild-type or mutant *UBE2G2*. (**C**) qRT-PCR analysis of *IFNB1*, *IFIT1*, and *CXCL10* expression in wild-type, *UBE2G2* knockdown, and *UBE2G2* knockdown with knockdown of *STING* HeLa cells after stimulation with diABZI for indicated times. (**D**) Immunoblot analysis of the indicated proteins in wild-type and *UBE2G2* knockdown HeLa cells after stimulation with diABZI for indicated times. (**E**) Immunoblot analysis of the indicated proteins in wild-type, *UBE2G2* knockdown, and *UBE2G2* knockdown with knockdown of *STING* HeLa cells after stimulation with diABZI for indicated times. (**F**) qRT-PCR analysis of *IFNB1*, *IFIT1*, and *CXCL10* expression in wild-type and *UBE2G2* knockdown HT1080 cells after stimulation with diABZI for indicated times. (G) Immunoblot analysis of the indicated proteins in wild-type and *UBE2G2* knockdown HT1080 cells after stimulation with diABZI for indicated times. Data are representative of three experiments with similar results. Bar graphs show the means ± SD. *P < 0.05, **P < 0.01, and ***P < 0.001.

To gain insights into the possible roles of UBE2G2 in innate immune responses, we examined the expression of various inflammatory cytokines in *UBE2G2* knockdown HeLa cells. Among these, the expression of ISGs (*CXCL10*, *IFIT1*, *ISG15*, *MX1*, *OAS1*) was significantly increased in *UBE2G2* knockdown HeLa cells compared to wild-type cells in the resting state (Fig. 5A). Reconstitution with wild-type *UBE2G2* or catalytically deficient (C89K) mutant of UBE2G2 in *UBE2G2* knockdown cells reduced the expression of ISGs back to levels observed in wild-type *UBE2G2* HeLa cells (Fig. 5B). After stimulation with the small-molecule agonist diABZI, *UBE2G2* knockdown significantly enhanced diABZI-induced transcription of downstream genes such as *IFNB1*, *IFIT1*, and *CXCL10*. However, *STING* knockdown in *UBE2G2* knockdown cells reversed this effect (Fig. 5C). Consistently, the phosphorylation of TBK1 following diABZI treatment was markedly increased in *UBE2G2* knockdown HeLa cells; this increase was abolished after *STING* knockdown (Fig. 5D and E). Similar results were observed in HT1080 cells, where *UBE2G2* knockdown enhanced the phosphorylation of TBK1 and the transcription of downstream antiviral genes (*IFNB1*, *IFIT1*, and *CXCL10*) following diABZI treatment (Fig. 5F and G). These experimental results indicate that the knockdown of *UBE2G2* can amplify STING-mediated innate immune response.

STING-induced autophagy is considered an evolutionarily conserved, nonclassical function independent of TBK1-IRF3 (5). Knockdown of *UBE2G2* can enhance STING-mediated innate immune response. Next, we aim to explore whether the knockdown of *UBE2G2* affects STING-induced autophagy. Interestingly, the knockdown of *UBE2G2* not only enhanced the STING-induced innate immune response but also increased LC3 lipidation. However, LC3 lipidation was abolished after the knockdown of *STING* (Fig. 4I, J, 5D, E, and G). These findings suggest that the knockdown of *UBE2G2* can enhance STING-induced autophagy.

### Knockdown of *Ube2g2* in mouse cells enhances STING-mediated innate immune response

**Fig 6:**
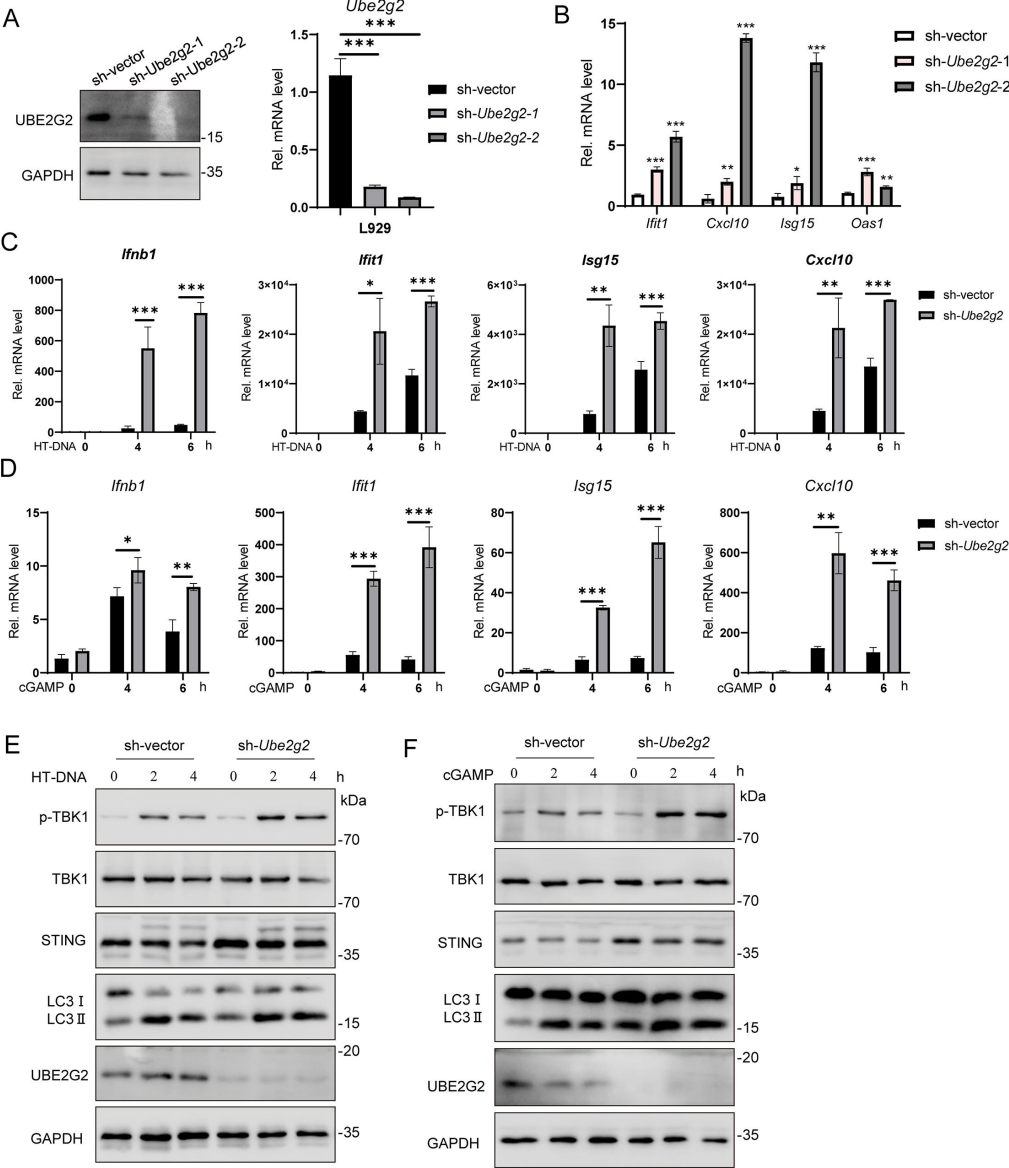
Knockdown of *Ube2g2* activates STING signaling in L929 cells. (**A**) Immunoblot analysis of Ube2g2 protein (left) and qRT-PCR analysis of *Ube2g2* mRNA level (right) in *Ube2g2* stably knockdown L929 cells. (**B**) qRT-PCR analysis of baseline ISGs (*Ifit1*, *Isg15*, *Oas1*, and *Cxcl10*) in wild-type *Ube2g2* and two independent *Ube2g2* knockdown L929 cell lines. (**C and D**) qRT-PCR analysis of *Ifnb1*, *Ifit1*, *Isg15*, and *Cxcl10* mRNA expression in wild-type and *Ube2g2* knockdown L929 cells after stimulation with HT-DNA (**C**) or cGAMP (1 µg/mL) (**D**) for indicated times. (**E and F**) Immunoblot analysis of the indicated proteins in wild-type and *Ube2g2* knockdown L929 cells after stimulation with HT-DNA (**E**) or cGAMP (1 µg/mL) (**F**) for indicated times. Data are representative of three experiments with similar results. Bar graphs show the means ± SD. *P < 0.05, **P < 0.01, and ***P < 0.001.

To investigate whether UBE2G2 regulates the STING-mediated innate immune response in mouse cell lines, we knocked down *Ube2g2* in L292 cells using lentivirus infection with shRNA. The knockdown effect was verified by qRT-PCR and Western blot (Fig. 6A). Notably, the knockdown of *Ube2g2* without any stimulation significantly increased the transcription of ISGs (Fig. 6B). Next, we stimulated the cGAS-STING pathway by transfecting L929 cells with HT-DNA or the second messenger 2′,3′-cGAMP. We found that the induction of *Ifnb1*, *Ifit1*, *Isg15*, and *Cxcl10* was significantly increased in *Ube2g2* knockdown L929 cells. Additionally, the phosphorylation of TBK1, induced by transfecting HT-DNA or cGAMP, was markedly enhanced in *Ube2g2* knockdown L929 cells. These data suggest that UBE2G2 plays a critical role in the STING-regulated innate immune response in mouse cell lines.

### AUP1 and UBE2G2 retain STING at the ER

**Fig 7:**
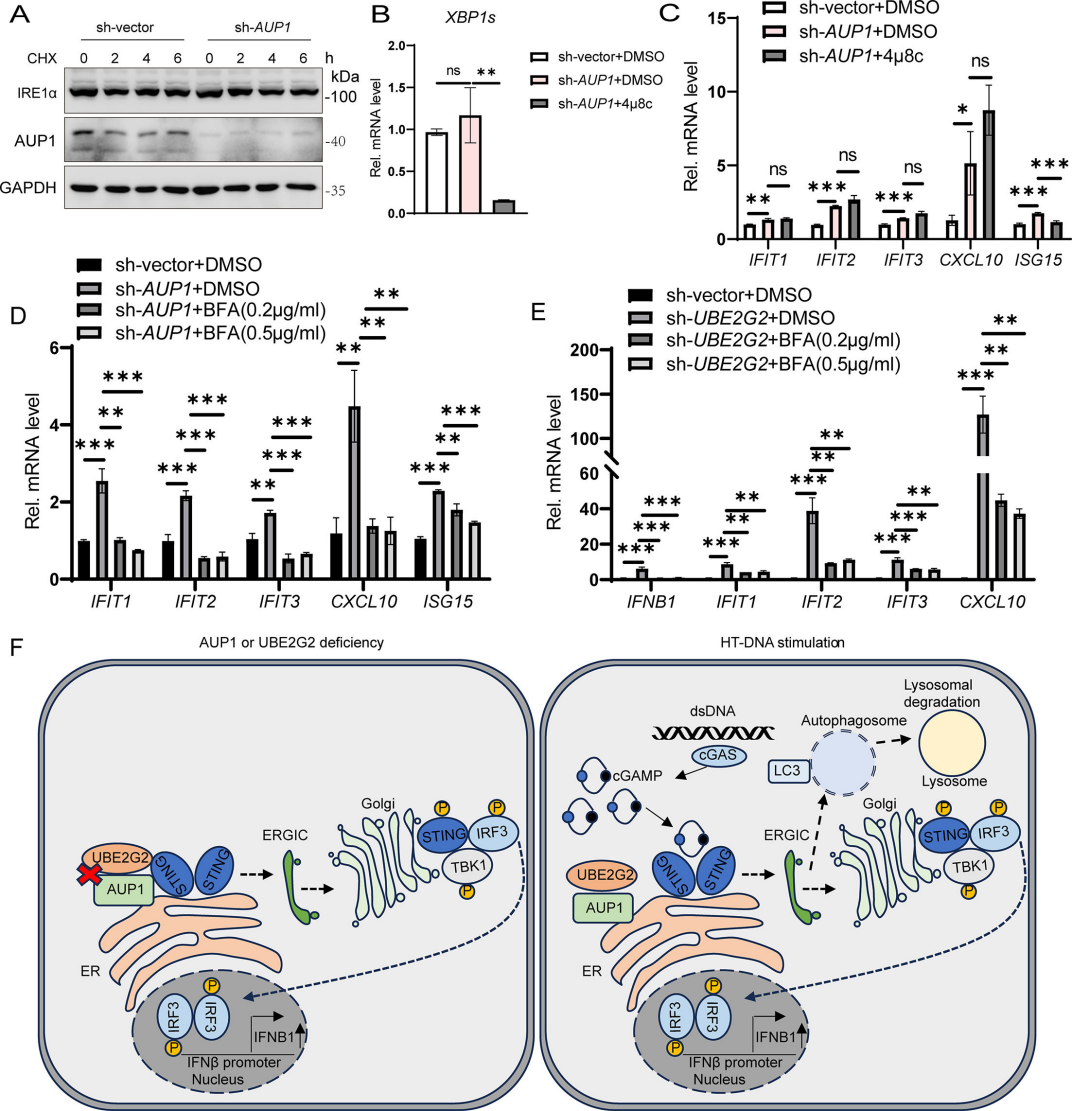
AUP1 and UBE2G2 retain STING at the ER. (**A**) Cells were treated with CHX (50 µg/mL) for the indicated times to inhibit protein synthesis and IRE1α degradation monitored by Western blot in wild-type and *AUP1* knockdown HeLa cells. (**B**) qRT-PCR analysis of spliced *XBP1* mRNA expression in wild-type and *AUP1* knockdown HeLa cells treated with DMSO or 4μ8c (50 µM) for 24 h. (**C**) qRT-PCR analysis of the baseline ISGs (*IFIT1*, *IFIT2*, *IFIT3*, *CXCL10*, and *ISG15*) in wild-type and *AUP1* knockdown HeLa cells treated with DMSO or 4μ8C (50 µM) for 24 h. (**D**) qRT-PCR analysis of the baseline ISGs (*IFIT1*, *IFIT2*, *IFIT3*, *CXCL10*, and *ISG15*) in wild-type and *AUP1* knockdown HeLa cells treated with DMSO or BFA for 6 h. (**E**) qRT-PCR analysis of the baseline *IFNB1* and ISGs (*IFIT1*, *IFIT2*, *IFIT3*, and *CXCL10*) in wild-type and *UBE2G2* knockdown THP1 cells treated with DMSO or BFA for 6 h. (**F**) A proposed model of AUP1 and UBE2G2 complex controls STING-mediated innate immunity. On the one hand, AUP1 and UBE2G2 interact with STING in the resting state, but when AUP1 or UBE2G2 deficiency occurs, STING exits from the ER and begins trafficking, inducing activation of downstream signaling. On the other hand, HT-DNA stimulation can disrupt the interaction of AUP1 and UBE2G2 complex with STING and promote the normal signaling of STING. Data are representative of three experiments with similar results. Bar graphs show the means ± SD. *P < 0.05, **P < 0.01, and ***P < 0.001.

STING signaling has been reported to be involved in ER stress and the unfolded protein response (UPR) (31, 32). ER-associated degradation (ERAD) and UPR are two closely linked pathways, with AUP1 playing a role in the ERAD pathway (28, 33). Therefore, we wondered whether AUP1 regulates STING signaling through ER stress and the UPR in the resting state. To assess whether the UPR is activated in *AUP1* knockdown cells, we measured the protein level of inositol-requiring enzyme 1α (IRE1α), an ER-resident sensor of ER stress or UPR (34). However, IRE1α protein levels did not change significantly in *AUP1* knockdown cells (Fig. 7A). Consistently, spliced *XBP1* mRNA, a marker for IRE1α activation, did not show significant changes in *AUP1* knockdown cells compared to wild-type cells (Fig. 7B). Next, we inhibited IRE1α activity with a small-molecule compound 4μ8C (35). While 4μ8C decreased the transcription level of spliced *XBP1*, it did not affect the transcription level of ISGs in *AUP1* knockdown cells in the resting state (Fig. 7B and C). Taken together, these data demonstrate that AUP1 has no effect on the UPR and IRE1α, and its effect on the STING pathway is UPR and IRE1α independent.

The exit of STING from the ER is a crucial step in its activation, and compound brefeldin A (BFA) can block this exit (18, 19). Therefore, we aim to investigate whether AUP1 and UBE2G2 anchor STING to the ER to restrict STING signaling. BFA treatment indeed reduced the transcription of ISGs in *AUP1* knockdown HeLa cells or *UBE2G2* knockdown THP1 cells in the resting state (Fig. 7D and E). Collectively, these data confirm that AUP1 and UBE2G2 can block STING trafficking in the resting state. AUP1 and UBE2G2 retain STING in ER at the resting state; however, when either AUP1 or UBE2G2 is deficient, STING can spontaneously activate downstream signals and promote the production of IFNβ. Furthermore, exogenous stimuli, such as dsDNA, can disrupt the interaction between AUP1 and STING, thereby facilitating normal downstream signaling transmission (Fig. 7F).

### *AUP1* deficiency enhances DNA virus-triggered signaling and inhibits virus replication *in vitro* and *in vivo*

**Fig 8:**
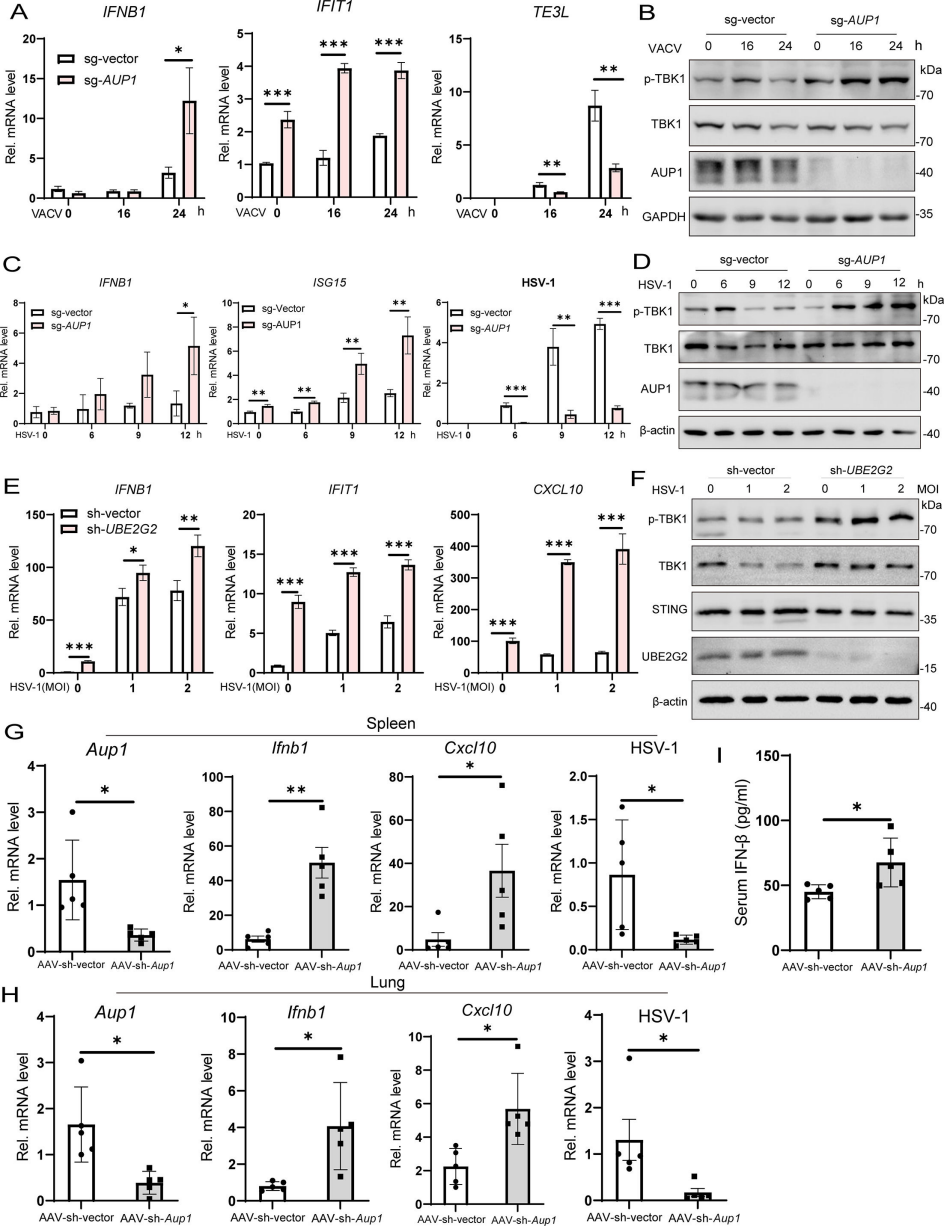
*AUP1* deficiency enhances DNA virus-triggered signaling and restricts viral infection *in vitro* and *in vivo.* (**A**) qRT-PCR analysis of *IFNB1*, *IFIT1*, and *TE3L* mRNA expression in wild-type and *AUP1* knockout HeLa cells infected with VACV (MOI = 0.1) for the indicated times. (**B**) Immunoblot analysis of the indicated proteins in wild-type and *AUP1* knockout HeLa cells infected with VACV (MOI = 0.1) for indicated times. (**C**) qRT-PCR analysis of mRNA abundance of the indicated genes in wild-type and *AUP1* knockout HeLa cells infected with HSV-1 (MOI = 1) for indicated times. (**D**) Immunoblot analysis of the indicated proteins in wild-type and *AUP1* knockout HeLa cells infected with HSV-1 (MOI = 1) for indicated times. (**E**) qRT-PCR analysis of mRNA abundance of the indicated genes in wild-type and *UBE2G2* knockdown THP1 cells infected with HSV-1 for 4 h. (**F**) Immunoblot analysis of the indicated proteins in wild-type and *UBE2G2* knockdown THP1 cells infected with HSV-1 for 4 h. (**G and H**) Two sets of mice were injected intravenously with AAV-sh-vector (1 × 10^12^ vg/mouse) or AAV-sh-*Aup1* (1 × 10^12^ vg/mouse) and kept under the same conditions for 4 weeks. Mice injected with AAV-sh-vector or AAV-sh-*Aup1* (*n*  =  5) were intravenously infected with HSV‐1 (0.5  ×  10^7^ PFU) for 24 h and then the lungs and spleens of the mice were subjected to real‐time PCR assays. (**I**) ELISA analyses of IFN-β from the sera of control and *Aup1* knockdown mice (*n*  =  5) after intravenous injection with HSV-1 (0.5  ×  10^7^ PFU) for 24 h. (**G–I**) Each symbol represents one independent biological replicate. Bar graphs show the means ± SD. *P < 0.05, **P < 0.01, and ***P < 0.001.

Viral nucleic acids act as classic pathogen-associated molecular patterns (PAMPs) that initiate the innate immune response. It has been demonstrated that cGAS recognizes cytosolic DNAs derived from various types of viruses, including DNA viruses, such as herpes simplex virus 1 (HSV-1) and vaccinia virus (VACV) (36). Here, we aimed to investigate whether AUP1 affects the innate immune response induced by DNA viruses by examining the expression of antiviral genes induced by VACV (Tian-Tan Strain) or HSV-1 in *AUP1* knockout HeLa cells. We found that induction of *IFNB1* and *IFIT1* mRNAs after VACV infection was significantly increased in *AUP1* knockout cells compared to their wild-type counterparts (Fig. 8A). Consistently, the phosphorylation of TBK1 induced by VACV was markedly increased in *AUP1*-deficient cells (Fig. 8B). Furthermore, the replication of VACV was remarkably inhibited in *AUP1* knockout cells (Fig. S4A and B). The production of virus was also severely inhibited in the supernatant of *AUP1* knockout cells, as determined by the plaque assay compared to control cells (Fig. S4C and D). In similar experiments, the induction of *IFNB1* mRNA and the phosphorylation of TBK1 following infection with another DNA virus, HSV-1 (F-strain), were significantly increased (Fig. 8C and D). Additionally, the replication of HSV-GFP was remarkably inhibited in *AUP1* knockout cells (Fig. S4E). Moreover, the knockdown of *UBE2G2* in THP1 cells significantly enhanced the HSV-1-induced innate immune response and limited viral replication (Fig. 8E and F; Fig. S4F). Next, to explore whether AUP1 influences the innate immune response induced by RNA viruses, we examined the phosphorylation levels of relevant proteins following infection with vesicular stomatitis virus (VSV) at different time points. We found no differences in the phosphorylation levels of IFN upstream IRF3 and downstream STAT1/STAT2 between *AUP1* knockout cells and wild-type counterparts (Fig. S4G). However, the replication of VSV was severely impaired in *AUP1* knockout cells (Fig. S4H). Interestingly, with the prolonged VSV infection time, AUP1 protein showed a tendency to accumulate (Fig. S4G). These results suggest that *AUP1* knockout enhances the innate immune response induced by DNA virus and inhibits the replication of the RNA virus VSV, although this inhibition appears to be independent of the IFN signaling pathway.

To gain insight into the importance of AUP1 in host defense against viral infection *in vivo*, we set out to suppress *Aup1* expression in mice with shRNAs delivered by adeno-associated virus serotype 9 (AAV9), which has a high transduction efficiency to many tissues (37). Two sets of mice were then injected intravenously with AAV-sh-vector or AAV-sh-*Aup1* and kept under the same conditions for 4 weeks. Next, HSV‐1 infection and antiviral responses were examined in a variety of organs of mice injected with AAV-sh-vector or AAV-sh-*Aup1*. Mice injected with AAV-sh-*Aup1* showed a considerably reduced expression of *Aup1* mRNA in the spleens and lungs (Fig. 8G and H). In the spleens and lungs, 24 h after HSV‐1 infection, compared to wild‐type mice, mice injected with AAV-sh-*Aup1* exhibited increased production of *Ifnb1* and *Cxcl10*, and reduced mRNA of HSV‐1 (Fig. 8G and H). In addition, enzyme-linked immunosorbent assay (ELISA) results indicated that the protein levels of IFN‐β in the serum were much higher in mice injected with AAV-sh-*Aup1* than in wild‐type mice 24 h after intravenous infection with HSV‐1 (Fig. 8I). These data demonstrated that *Aup*1 deficiency can enhance DNA virus-induced innate immune response and restrict viral infection *in vivo*.

### AUP1 is upregulated by VSV infection and impairs the replication of VSV by regulating lipid accumulation

**Fig 9:**
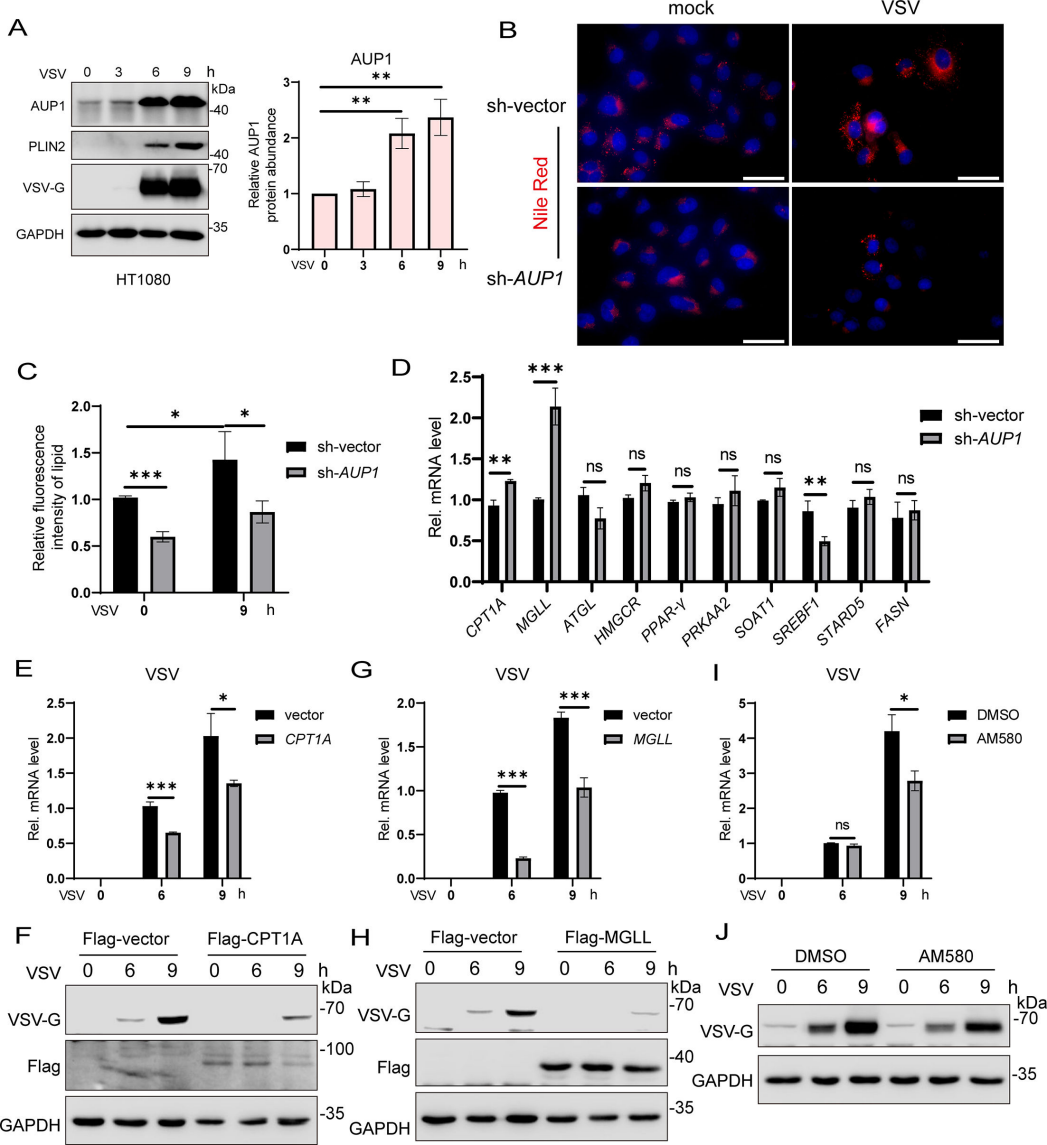
VSV infection induces AUP1 protein accumulation, and knockdown of *AUP1* alters lipid metabolism genes. (**A**) Immunoblot analysis of the indicated proteins at different time intervals upon VSV (MOI = 1) infection in HT1080 cells (left) and densitometric analyses to quantitate increased expression of AUP1 (right). GAPDH was used as an internal control. (**B and C**) LDs (stained with Nile Red) visualized using confocal imaging. Scale bar represents 50 µm. The fluorescence intensity of lipid was analyzed with ImageJ. (**D**) qRT-PCR analysis of mRNA expression levels of various lipid metabolism-associated factors in wild-type and *AUP1* knockdown HeLa cells. (**E and F**) HeLa cells were transfected with vector or Flag-CPT1A for 24 h, followed by infection with VSV (MOI = 0.1) for indicated time. The mRNA and protein levels of VSV were determined by qRT-PCR and Western blot. (**G and H**) HeLa cells were transfected with vector or Flag-MGLL for 24 h, followed by infection with VSV (MOI = 0.1) for indicated times. The mRNA and protein levels of VSV were determined by qRT-PCR and Western blot. (**I and J**) HeLa cells were infected with VSV (MOI = 1) and then treated with DMSO or AM580 (20 µM) for indicated times. The mRNA and protein levels of VSV were determined by qRT-PCR and Western blot. Data are representative of three experiments with similar results. Bar graphs show the means ± SD. *P < 0.05, **P < 0.01, and ***P < 0.001.

AUP1 is a lipid droplet protein, and confocal microscopy revealed that AUP1 (mCherry) co-localized with the LD (BODIPY493/503) (Fig. S5A). A previous report indicated that dengue virus infection leads to altered AUP1 protein abundance (38). Our result showed that VSV infection appears to increase AUP1 protein levels in HeLa cells (Fig. S4G). Actually, as the duration of VSV infection increased, AUP1 protein level also gradually accumulated, and the LD marker protein PLIN2 also showed a gradual increase, which is a common phenomenon (Fig. 9A; Fig. S5C). However, VSV infection did not affect *AUP1* mRNA levels (Fig. S5B). Furthermore, the knockdown of *AUP1* significantly inhibited VSV replication (Fig. S5D and E). These data suggest that the accumulation of AUP1 protein induced by VSV infection occurs after translation, and AUP1 can regulate VSV replication.

Cellular LDs can participate in several processes of viral proliferation such as attachment, viral genome replication, and viral budding. Viruses can hijack lipid biogenesis pathways and use intracellular lipids to promote viral proliferation. Therefore, we wanted to explore whether AUP1 affects VSV replication by regulating lipid accumulation. In fact, lipid accumulation in *AUP1* knockdown cells was attenuated by immunofluorescence assays, and although VSV increased intracellular lipid accumulation, *AUP1* knockdown significantly inhibited virus-induced lipid accumulation (Fig. 9B and C). AUP1 regulates the expression of lipid metabolism genes, thus affecting the synthesis of LDs (39). To verify this, we detected the expression of several important lipid metabolism genes in *AUP1* knockdown cells (Fig. 9D) (39–41). As shown in Fig. 9D, among these genes whose mRNA expression levels were affected by *AUP1* knockdown, *CPT1A* and *MGLL* were significantly increased, while *SREBF1* was decreased. However, the expression of these genes did not change in *UBE2G2* knockdown cells (Fig. S5F). Next, we wanted to investigate whether these lipid metabolism genes, specifically *CPT1A*, *MGLL*, and *SREBF1*, regulate VSV replication. Notably, the overexpression of *CPT1A* or *MGLL* can significantly inhibit VSV replication (Fig. 9E through H). Moreover, cells treated with AM580, an inhibitor of SREBF1, followed by VSV infection, showed decreased VSV mRNA and protein levels (Fig. 9I and J). In addition, VSV infection did not affect *CPT1A*, *MGLL*, and *SREBF1* expression (Fig. S5G). These data suggest that AUP1 can regulate the expression of lipid metabolism genes, affect lipid accumulation, and thus regulate viral replication.

## DISCUSSION

STING, a key signaling adaptor protein in DNA-sensing pathways, localizes to the ER membrane in the resting state. Typically, STING activation requires the binding of the ligand cGAMP, which triggers a series of subsequent reactions. However, STING can also be activated in a ligand-independent manner, during which it spontaneously exits the ER and traffics to the Golgi apparatus (18, 19). This spontaneous activation of STING can lead to immune disorders and the development of autoimmune diseases. Therefore, preventing the abnormal activation of STING is essential for maintaining immune homeostasis. Nonetheless, it remains unclear how STING’s resting state is maintained. Here, we report the identification of the AUP1-UBE2G2 complex as a key suppressor of STING innate immunity.

AUP1 is involved in several pathways, including the quality control of misfolded proteins in the ER and LD accumulation (23, 24, 42). In our study, multiple lines of evidence substantiate the key role of AUP1 in STING-mediated innate immune responses. First, the deficiency of endogenous AUP1 specifically enhances dsDNA-induced cGAS-STING signaling, promoting the transcription of IFNs and ISGs, but it does not affect the poly(I:C)-induced RIG-I/MDA5 signaling pathway. Second, AUP1 and STING interact closely with one another, as evidenced by multiple functional regions of AUP1 and STING interacting with the full lengths of each protein at varying intensities. The close interaction between AUP1 and STING indicates that AUP1 may be crucial for regulating STING signaling. Third, the absence of endogenous AUP1 boosts STING signaling in the resting state and significantly increases the expression of several ISGs. Fourth, the addition of STING trafficking inhibitor BFA reduces the increase of ISGs induced by *AUP1* knockdown in the resting state. Because BFA inhibits STING trafficking to ERGIC, the increase in ISGs resulting from *AUP1* knockdown is diminished. Mechanistically, AUP1 may interact with STING to retain it in the ER, preventing STING trafficking to ERGIC and thereby avoiding continuous activation of downstream signaling in the resting state. Fifth, deficiency of endogenous AUP1 significantly inhibits DNA virus replication and enhances the DNA virus-induced innate immune response *in vitro* and *in vivo*. Notably, AUP1 interacts strongly with STING containing only four transmembrane domains, but weakly with STING containing ligand-binding domains, indicating that AUP1 may retain it at the ER mainly by binding to STING’s transmembrane region. Therefore, we hypothesized that AUP1 primarily retains STING in the ER by binding to STING’s transmembrane domain, thereby restricting STING’s trafficking to ERGIC.

UBE2G2 is an E2 that acts as a cofactor of AUP1 and has been reported to interact with multiple E3 ubiquitin ligases, such as HRD1 and AMFR, which are involved in substrate degradation (22, 26, 28). However, our current study reveals that UBE2G2 regulates STING’s resting state activation independently of its enzymatic activity (C89), because reconstitution with the catalytically deficient (C89K) mutant of UBE2G2 reduced ISGs expression in *UBE2G2* knockdown cells, suggesting that UBE2G2 may not regulate STING signaling through the ERAD pathway. A previous study has reported that basal levels of autophagosomes (LC3-II) are significantly higher in UBE2G2^−/−^ cells (43), and we confirm this phenomenon here. Although *STING* knockdown in *UBE2G2* knockdown cells reverses dsDNA or diABZI-induced LC3-II accumulation, it does not reduce resting LC3-II levels. This may indicate that the increase of basal LC3-II after *UBE2G2* knockdown is not related to STING spontaneous activation. While UBE2G2 participates in STING signaling regulated by AUP1, the specific mechanism remains unclear. AUP1 interacts with STING, but AUP1 lacking the G2BR domain interacts with STING weakly (Fig. 2F). It has been reported that the G2BR domain of AUP1 recruits its cofactor UBE2G2 to ER and enhances UBE2G2 protein stability (23, 28). Hence, we speculate that UBE2G2 may strengthen the interaction between AUP1 and STING, anchoring STING firmly in ER during the resting state.

It is interesting to note that *AUP1* deficiency markedly inhibited the replication of the RNA virus VSV, possibly related to the increase of antiviral ISGs in the resting state. However, *AUP1* deficiency did not cause significant changes in VSV-induced phosphorylated proteins compared with controls. This suggests that AUP1 may regulate viral replication through mechanisms that do not solely depend on modulating the innate immune response. Additionally, infection with VSV led to an accumulation of AUP1 protein levels in various cell types, while no significant accumulation was observed during VACV and HSV-1 infections. We hypothesized that this accumulation of AUP1 protein was due to VSV infection increasing the abundance of LDs, as indicated by induced accumulation of the LD protein marker PLIN2. Increasing data suggest that LDs also play an important role in viral proliferation, highlighting their potential as a therapeutic target for viruses (40, 41). Previous reports have indicated that AUP1 can regulate lipid metabolism and induce lipid accumulation (23, 39). Here, we confirm that AUP1 regulates the transcription of genes associated with lipid metabolic pathways such as *CPT1A*, *MGLL*, and *SREBF1*. CPT1A is a key fatty acid beta oxidation enzyme, which can degrade intracellular lipid accumulation (44). As the key enzyme of lipolysis, *MGLL* deficiency results in lipid overload in TAMs (45). SREBF1 promotes cellular lipid synthesis; many studies have confirmed that SREBF1-mediated lipid metabolism is a prerequisite for cellular lipid homeostasis and efficient viral replication (41). Therefore, we propose that VSV might exploit AUP1 to regulate lipid accumulation and promote its own replication.

In summary, our study reveals that AUP1 acts as a negative regulator of STING signaling and identifies that AUP1 regulates lipid metabolism and influences viral replication. This research may provide a conceptual strategy for enhancing antiviral therapy or treating autoimmune diseases.

## MATERIALS AND METHODS

### Mice

C57BL/6 mice (6–8 weeks of age, male) were used for experiments unless otherwise specified. All mice were bred and kept under specific pathogen-free conditions in the animal care facility of Hunan University. Mice were infected with HSV-1 intravenously. Mice were injected intravenously with AAV9-sh-*Aup1* (GenePharma, Shanghai, China). The target sequences of shRNAs are shown in Table S2.

### Cell culture

HeLa (National Collection of Authenticated Cell Cultures), HEK293T (National Collection of Authenticated Cell Cultures), HT1080, and L929 cells were cultured in Dulbecco’s modified Eagle’s medium (DMEM; Gibco). THP1 cells were grown in RPMI 1640 (Gibco) supplemented with β-mercaptoethanol (0.05 mM). PMA‐THP1 cells refers to THP1 cells that were pretreated with 185 ng/mL PMA for 24 h. All cells were supplemented with 10% fetal bovine serum (FBS; Gibco), penicillin (100 U mL^−1^), and streptomycin (100 mg mL^−1^) (Gibco) under humidified conditions with 5% CO_2_ at 37°C.

### Viruses

The viruses used in this study include herpes simplex virus 1 (F strain, ATCC VR-733), herpes simplex virus 1-GFP (Kos strain), caccinia virus (Tian-Tan strain), caccinia virus-GFP (Tian-Tan strain), and vesicular stomatitis virus (Indiana strain). The vesicular stomatitis virus-GFP was kindly shared by Hai-Zhen Zhu (Hunan University, Changsha, Hunan, China). Virus was propagated and ampliﬁed in Vero cells. Supernatants were harvested and clariﬁed by centrifugation. Viral titer was determined by plaque assay in Vero cells.

### Antibodies and reagents

The primary antibodies used include anti-AUP1 (Proteintech, 13726-1-AP, immunoblot 1:1,000), anti-UBE2G2 (ABclonal, A10408, immunoblot 1:1,000), anti-phospho-TBK1/NAK (Ser172) (D52C2) (CST, 5483, immunoblot 1:1,000), anti-TBK1/NAK (D1B4) rabbit mAb (CST, 3504, immunoblot 1:1,000), anti-phospho-IRF-3 (Ser396) (4D4G) (CST, 4947, immunoblot 1:1,000), anti-IRF3 (Proteintech, 11312-1-AP, immunoblot 1:3,000), anti-phospho-STING (Ser366) (E9A9K) (CST, 50907, immunoblot 1:1,000), anti-STING (Proteintech, 19851-1-AP, immunoblot 1:1,000), anti-phospho-STAT1 (Tyr701) (Proteintech, 28979-1-AP, immunoblot 1:1,000), anti-STAT1 (Proteintech, 10144-2-AP, immunoblot 1:1,000), anti-phospho-Stat2 (Tyr690) (D3P2P) (CST, 88410, immunoblot 1:1,000), anti-LC3A/B (CST, 4108, immunoblot 1:1,000), anti-V5 tag (Invitrogen, R960-25), anti-DYKDDDDK tag (Proteintech, 20543-1-AP, immunoblot 1:5,000), anti-DYKDDDDK tag (Proteintech, 66008-4-Ig, immunoblot 1:5,000), anti-IRE1α (Proteintech, 27528-1-AP, immunoblot 1:3,000), anti-HA tag (Proteintech, 51064-2-AP, immunoblot 1:5,000), anti-VSV-G tag (Abcam, ab183497, immunoblot 1:1,000), and anti-ADRP/perilipin-2 (Proteintech, 15294-1-AP, immunoblot 1:1,000). Donkey anti-rabbit IgG (H+L) Alexa Fluor-488 (Thermo Fisher Scientific) and goat anti-mouse IgG (H+L) Alexa Fluor-555 were used as secondary antibodies. The following reagents were also used: HT-DNA (Sigma-Aldrich, D6898), poly(I:C) (InvivoGen, tlrl-pic), Lipofectamine 2000 reagent (Invitrogen, 11668019), 2′,3′-cGAMP (InvivoGen, tlrl-nacga23-02), diABZI (Selleck, S8796), brefeldin A (Selleck, S7046), cycloheximide (Selleck, S7418), 4μ8c (MedChemExpress, HY-19707), AM580 (TargetMOI, T5854), and Nile Red (HY-D0718, MedChemExpress).

### ELISA

The serum of mice was collected for measurement of IFN‐β (D721013, Sangon Biotech) by ELISA.

### Real-time PCR assay

RNA was extracted following the manufacturer’s protocol (RNA isolater Total RNA Extraction Reagent, Vazyme). RNA was reverse transcribed using the One-step gDNA Removal (TransGen Biotech) and was analyzed by qRT-PCR using the ChamQ Universal SYBR qPCR Master Mix (Vazyme). The qPCR reactions were run on a CFX96 Real-Time System (Bio-Rad). *GAPDH* was used as a housekeeping gene for normalization. The primers used for real-time PCR are listed in Table S1.

### Plasmids

For CRISPR-Cas9 plasmids, single-guide RNA (sg-RNA) targeting *AUP1* was designed using the web tool CRISPR (46). The *AUP1* single-guide RNAs were designed, synthesized, and cloned into the pSpCas9(BB)-2A-Puro (PX459) (Addgene Plasmid #48139). shRNA targeting *AUP1*, *UBE2G2*, and *STING* were designed, synthesized, and cloned into the pLKO.1-Puro. Single-guide RNA sequences and shRNA sequences are listed in Table S2.

*AUP1*, *UBE2G2*, and *STING* cDNAs were synthesized from the total cellular RNA isolated from HeLa cells by standard reverse transcription-PCR (RT-PCR). Subsequently, they were cloned into the pcDNA3.1a-V5-vector or p3×FLAG-CMV-vector. Multiple domains of AUP1 and STING were ampliﬁed from the templates of full-length AUP1 and STING, respectively, which were then cloned into the p3×FLAG-CMV-vector. pCDNA3.1-3×HA-TMEM173 (P8966) was obtained from MiaoLingBio, China. All constructs were confirmed by sequencing.

### Stimulation of cells

Cells were treated with 2.5 µM diABZI and collected at the indicated time points. For HT-DNA, poly(I:C) and cGAMP were transfected using Lipofectamine 2000 according to the manufacturer’s protocol.

### Generation of CRISPR-Cas9-mediated stably knockout cell lines

HeLa AUP1 KO cells were generated using CRISPR-Cas9 technology. In brief, HeLa cells were plated in 6-well culture plates at about 80% confluency and were transfected. Per well, 6 µL Lipofectamine 2000 and 2 µg plasmid DNA were each diluted in 100 µL OptiMEM (Gibco, 31985070), mixed, incubated for 5 min and then incubated together for 20 min, and added on top of the well. The next day, the culture medium was replaced, and cells were put under puromycin (2 µg mL^−1^) selection for 3 days. The single-cell clones were selected and identified by Western blotting.

### Lentivirus production and infection

HEK293T cells plated on 6 cm dishes were transfected with 750 ng of packaging plasmid psPAX2, 250 ng of envelope plasmid pMD2G, and 1 µg of the targeted plasmid encoding shRNA (pLKO.1-Puro) using polyethylenimine (PEI). After 12 h of transfection, the cells were washed and cultured with fresh medium. The supernatants were collected at 24, 48, and 72 h post-transfection and clariﬁed by ﬁltration through 0.45 µm syringe ﬁlters. For cell infection, the cells were plated in 12-well plates and infected with lentivirus for 72 h. The lentivirus-infected cells were selected for at least 5 days using puromycin (2 mg/mL) and subjected to the required assays.

### Western blotting and immunoprecipitation

Cells were washed with PBS (phosphate-buffered saline) and lysed with RIPA buffer (Thermo Fisher Scientiﬁc) supplemented with 1% protease inhibitor cocktail and phosphatase inhibitor cocktail (TargetMol, USA). The lysates were incubated on ice for 30 min and centrifuged at 12,000 × *g* for 15 min at 4℃. Supernatants were boiled with 5× loading buffer at 98°C for 6 min. Proteins were resolved on SDS-PAGE gels and transferred to polyvinylidene diﬂuoride (PVDF) membranes (Merck Millipore). The PVDF membranes were then blocked with 5% skim milk, sequentially incubated with primary and secondary antibodies. Membranes were washed with Tris-buffered saline with Tween (TBST), and the bound antibodies were detected using SuperSignal West Pico PLUS Luminol/Enhancer (Thermo Scientific). For immunoprecipitation, cells were seeded into 6-well plates and were transfected with the indicated plasmids. After 24 h of transfection, cells were lysed with 250 µL Pierce IP Lysis Buffer (Thermo Fisher Scientiﬁc) supplemented with 1% protease inhibitor cocktail on ice for 30 min and centrifuged at 12,000 × *g* at 4°C for 15 min. Supernatants (200 µL) were transferred into new tubes and mixed with anti-Flag or IgG at 4°C overnight on a rotator. The supernatants containing the antibody were mixed with 25 µL bead (Santa Cruz Biotechnology, sc-2003) and incubated for 6 h at 4°C on a rotator. After six washes with cold 1× PBS, the beads were boiled with 1× loading buffer for 8 min at 98°C. Samples were loaded into gel after a short centrifugation, and this was followed by SDS-PAGE and immunoblotting analysis.

### Confocal microscopy

HeLa cells seeded into a confocal dish were transfected with p3×Flag-CMV-AUP1 for 24 h in complete medium. For fixed-cell microscopy, cells were fixed with 4% paraformaldehyde for 20 min, permeabilized with 0.2% Triton X-100 in PBS buffer, and blocked with 3% bovine serum albumin (BSA) in PBS at room temperature for 1 h. Cells were incubated with primary antibody overnight at 4℃. After incubation of primary antibodies, cells were incubated with ﬂuorescence-labeled secondary antibodies at room temperature for 2 h. Nuclei were stained with Hoechst 3342 for 30 min. Imaging of the cells was carried out using Leica laser scanning confocal microscopy. Co-localization was analyzed by ImageJ.

HeLa cells seeded into a confocal dish were transfected with p3×Flag-CMV-mCherry-AUP1 for 24 h in complete medium. For fixed-cell microscopy, cells were fixed with 4% paraformaldehyde for 30 min, and then lipid droplets were stained with Bodipy493/503 for 30 min. Nuclei were stained with Hoechst 3342 for 30 min. Imaging of the cells was carried out using Leica laser scanning confocal microscopy.

### Plaque assay

Serial dilutions of supernatants from infected cells were added to HeLa cells at 37℃. After 1.5 h of adsorption, cells were washed, and 1% agarose media were added on top of the virus-infected HeLa cells. After 3–6 days of incubations, the cells with plaques were ﬁxed with 4% paraformaldehyde for 30 min at room temperature and were stained with 0.1% crystal violet.

## SUPPLEMENTAL MATERIAL

Supplemental material mbio.00602-25-s0001.docx Fig. S1 to S5; Tables S1 and S2.

ASM does not own the copyrights to Supplemental Material that may be linked to, or accessed through, an article. The authors have granted ASM a non-exclusive, world-wide license to publish the Supplemental Material files. Please contact the corresponding author directly for reuse.
