# Supplementary material for: AUP1 and UBE2G2 complex targets STING signaling and regulates virus-induced innate immunity
Source: mBio. 2025 Apr 16;16(5):e00602-25. doi: 10.1128/mbio.00602-25 (PMC12077101; doi:10.1128/mbio.00602-25)
Supplement: Supplemental material — Fig. S1 to S5; Tables S1 and S2. [file mbio.00602-25-s0001.docx]

**Supplementary Figures**


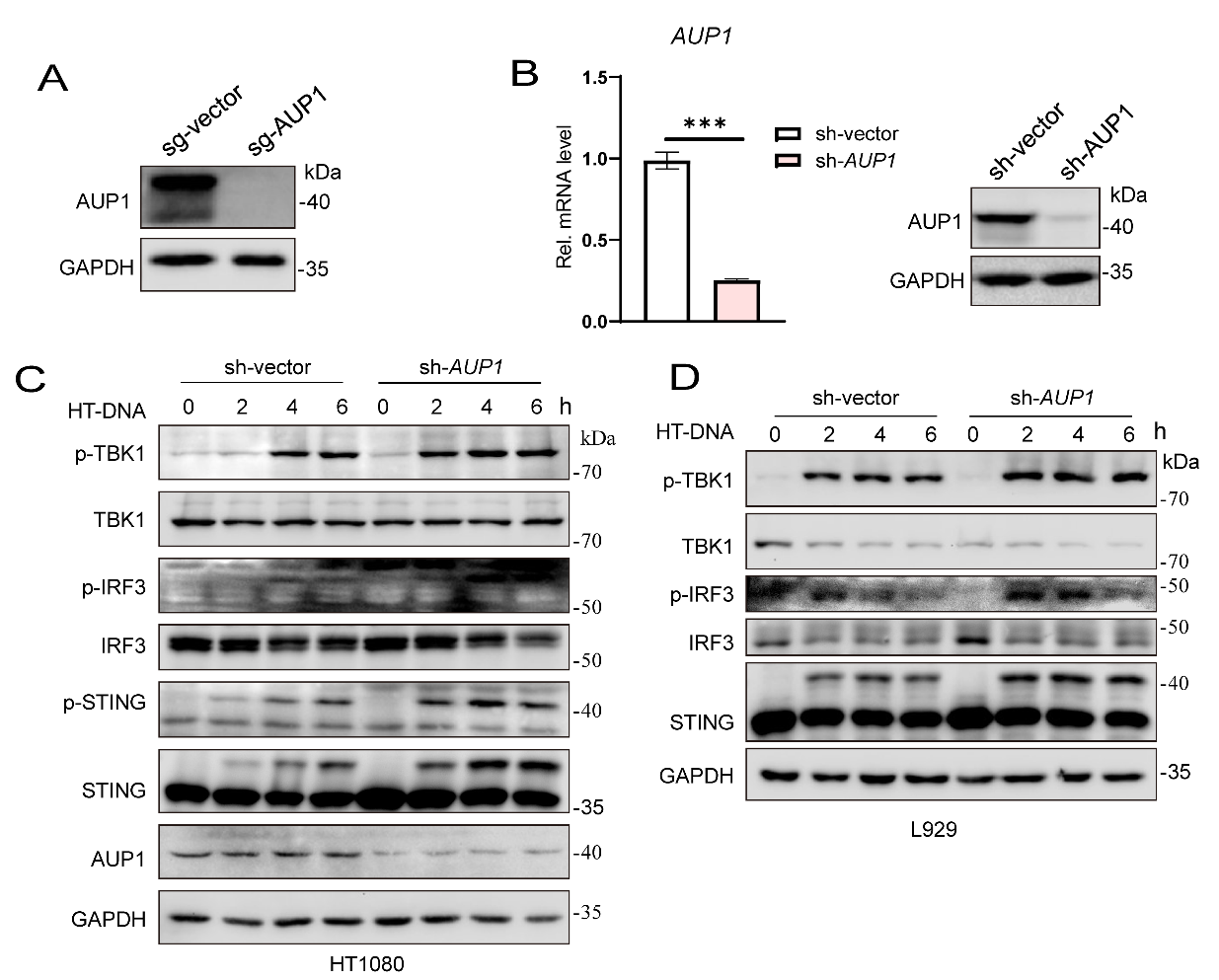


**Fig. S1:** ***AUP1* deficiency promotes cGAS-STING signaling pathway.** (A) *AUP1* knockout HeLa cells were generated using CRISPR-Cas9. Immunoblot analysis of AUP1 protein in wild-type (sg-vector) or *AUP1*-deficient (sg-*AUP1*) HeLa cells. (B) qRT-PCR analysis the level of *AUP1* mRNA (left) and immunoblot analysis of AUP1 protein (right) in *AUP1* knockdown HeLa cells. (C and D) Immunoblot analysis of the indicated proteins in wild-type and *AUP1* stably knockdown HT1080 or L929 cells transfected with the HT-DNA for indicated times. Data are representative of three experiments with similar results. Bar graphs show the means “±” SD. **P* < 0.05, ** *P* < 0.01, and *** *P* < 0.001.

**
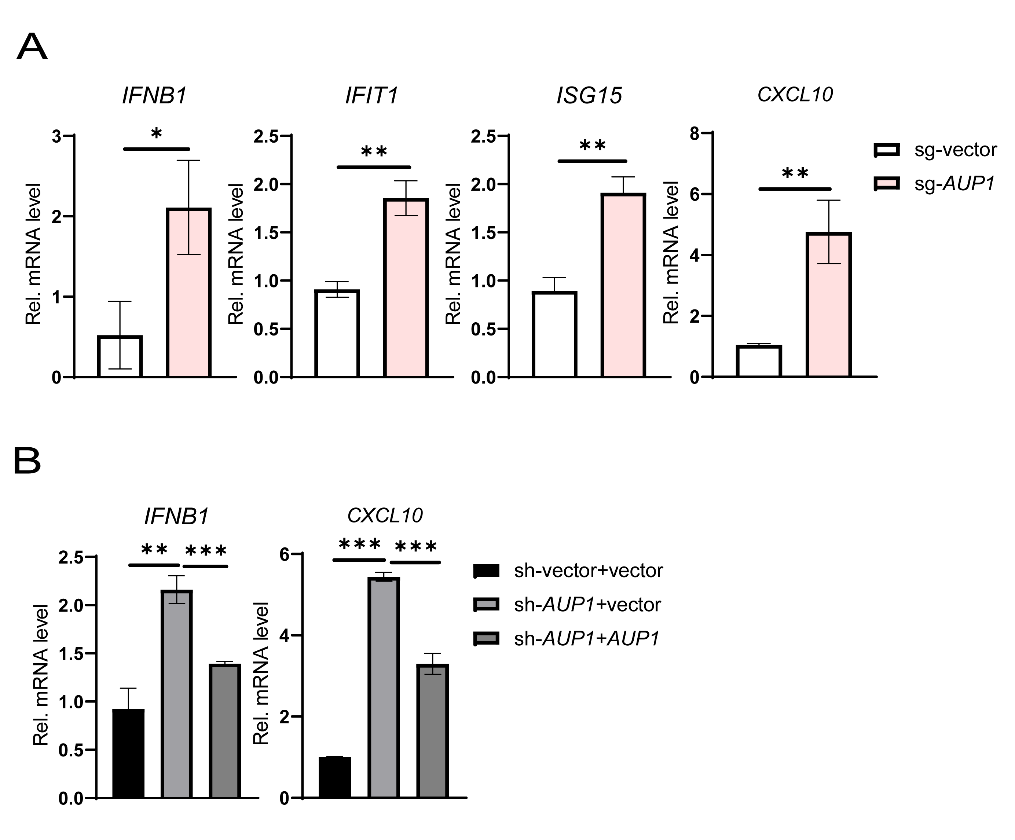
**

**Fig. S2:** **Knockout of *AUP1* promotes STING signaling.** (A) qRT-PCR analysis of the mRNA expression of resting-state *IFNB1* and ISGs (*IFIT1*, *ISG1 5*, and CXCL10) in *AUP1* knockout and wild-type cells. (B) qRT-PCR analysis of the baseline *IFNB1* and *CXCL10* expression in wild-type, *AUP1* knockdown and *AUP1* knockdown cells stably expressing wild-type *AUP1*. Data are representative of three experiments with similar results. Bar graphs show the means “±” SD. **P* < 0.05, ** *P* < 0.01, and *** *P* < 0.001.


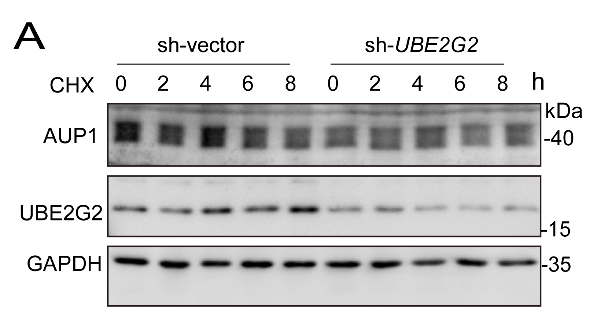


**Fig. S3: UBE2G2 is required for AUP1 to regulate STING signaling.** (A) Immunoblot analysis in wild-type and *UBE2G2* knockdown HeLa cells treated with CHX (50 μg/ml) for the indicated times.


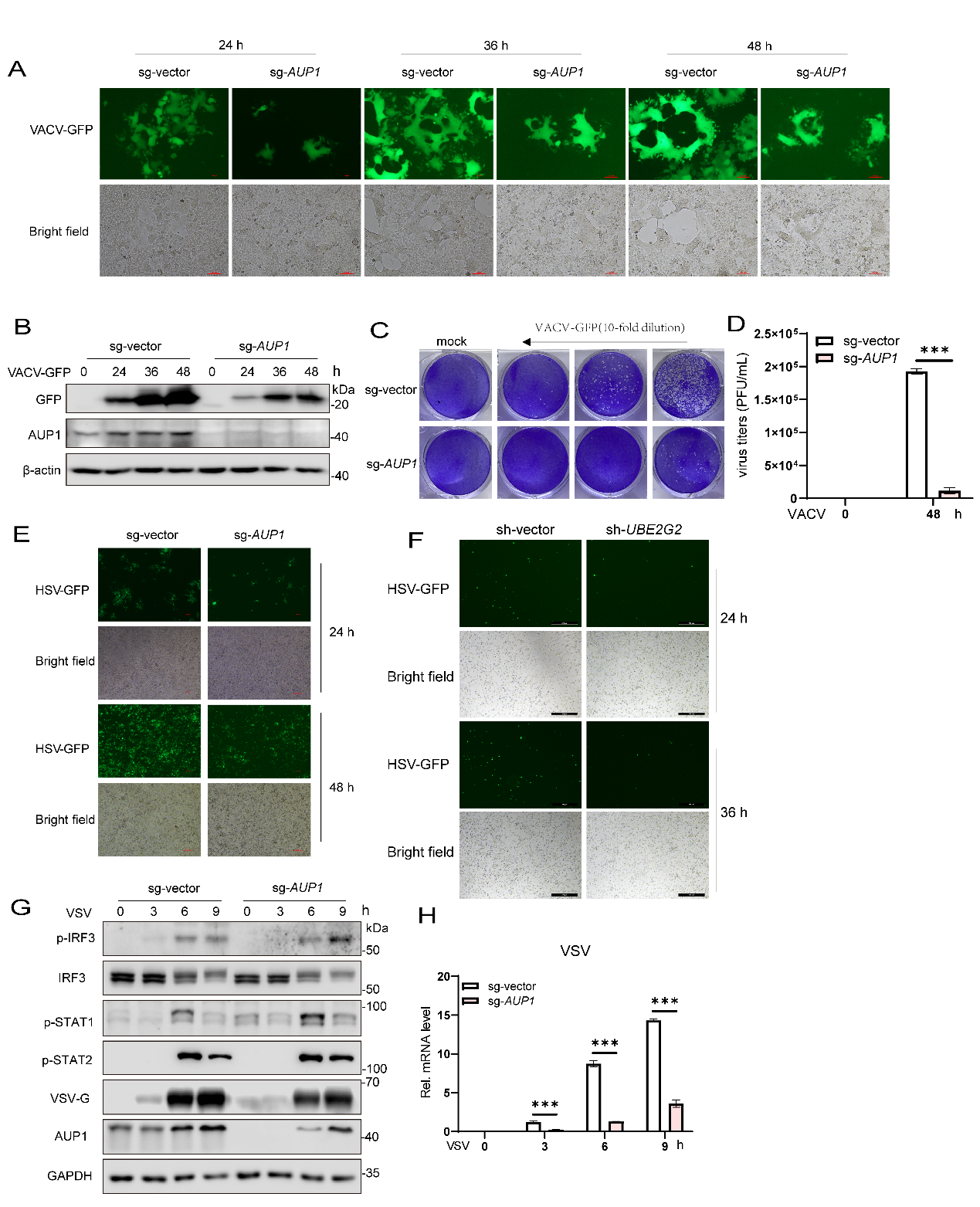


**Fig. S4:** ***AUP1* or *UBE2G2* deficiency enhances DNA virus-triggered signaling and inhibits virus replication.** (A) VACV-GFP replication in wild-type or *AUP1* knockout HeLa cells after infection for indicated times was visualized by fluorescence microscopy. (B) Immunoblot analysis of the indicated proteins in wild-type and *AUP1* knockout HeLa cells infected with VACV-GFP (MOI=0.01) for indicated times. (C) Representative images of plaque assays performed with supernatants collected from wild-type and *AUP1* knockout HeLa cells after being challenged with VACV (MOI=0.1, 48 h). (D) Virus particles produced in supernatants were quantitated using plaque assay from wild-type and *AUP1* knockout HeLa cells at 48 h post infection. PFU, plaque-forming units. (E) HSV-GFP (MOI=0.01) replication in wild-type or *AUP1* knockout HeLa cells after infection for indicated times was visualized by fluorescence microscopy. (F) HSV-GFP (MOI=0.001) replication in wild-type or *UBE2G2* knockdown THP1 cells after infection for indicated times was visualized by fluorescence microscopy. (G) Immunoblot analysis of the indicated proteins in wild-type and *AUP1* knockout HeLa cells infected with VSV (MOI=1) for indicated times. (H) qRT-PCR analysis of viral mRNA expression in wild-type and *AUP1* knockout HeLa cells infected with VSV (MOI=1) for indicated times. Data are representative of three experiments with similar results. Bar graphs show the means “±” SD. **P* < 0.05, ** *P* < 0.01, and *** *P* < 0.001.


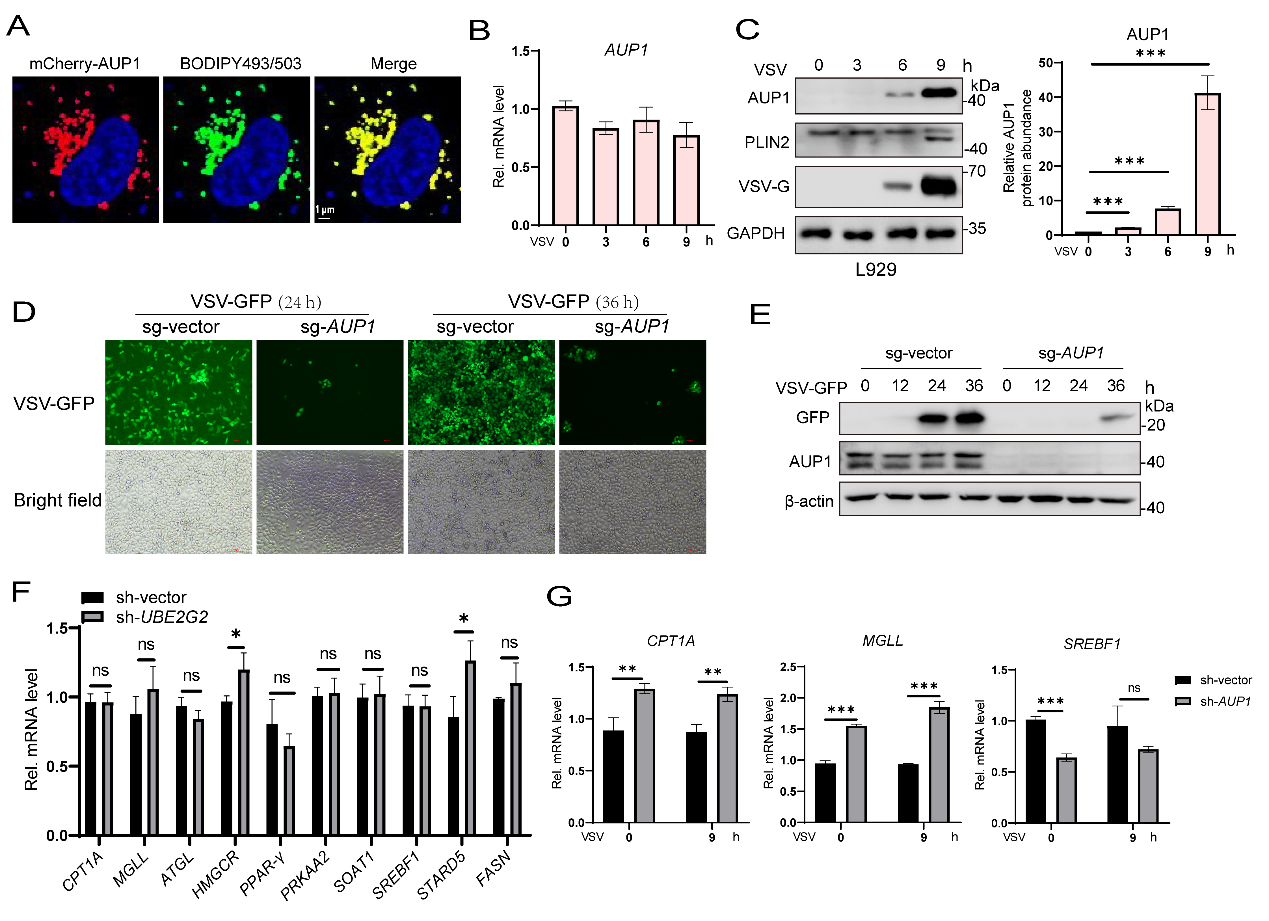


**Fig. S5: AUP1 is upregulated by VSV infection and impairs the replication of VSV by regulating lipid accumulation.** (A) LDs (stained with BODIPY) and mCherry-AUP1 visualized using confocal imaging. Scale bar represents 1 μm. (B) qRT-PCR analysis of mRNA abundance of *AUP1* at different time intervals upon VSV infection. (C) Immunoblot analysis of the indicated proteins at different time intervals upon VSV (MOI=1) infection in L929 cells (left) and densitometric analyses to quantitate increased expression of AUP1 (right). GAPDH was used as an internal control. (D) VSV-GFP replication in wild-type or *AUP1* knockout HeLa cells after infection for indicated times was visualized by fluorescence microscopy. (E) Immunoblot analysis of the indicated proteins in wild-type and *AUP1* knockout HeLa cells infected with VSV-GFP (MOI=0.0001) for indicated times. (F) qRT-PCR analysis of mRNA expression levels of various lipid metabolism-associated factors in wild-type and *UBE2G2* knockdown HeLa cells. (G) qRT-PCR analysis of mRNA expression levels of lipid metabolism-associated factors (*CPT1A*, *MGLL*, and *SREBF1*) in wild-type and *AUP1* knockdown HeLa cells infected with VSV (MOI=1) for indicated times. Data are representative of three experiments with similar results. Bar graphs show the means “±” SD. **P* < 0.05, ** *P* < 0.01, and *** *P* < 0.001.

**Supplementary Table 1: Primers for Real-time PCR**

| Primer designation | Primer sequence |
| --- | --- |
| Human-*IFNB1* (F) | 5’-CATTACCTGAAGGCCAAGGA-3’ |
| Human- *IFNB1* (R) | 5’-CAGCATCTGCTGGTTGAAGA-3’ |
| Human-*IFIT1* (F) | 5’-ATTTACAGCAACCATGAGTACAAA-3’ |
| Human-*IFIT1* (R) | 5’-TCCCACACTGTATTTGGTGTC-3’ |
| Human-*IFIT2* (F) | 5’-AAGCACCTCAAAGGGCAAAAC-3’ |
| Human-*IFIT2* (R) | 5’-TCGGCCCATGTGATAGTAGAC-3’ |
| Human-*IFIT3* (F) | 5’-TCAGAAGTCTAGTCACTTGGGG-3’ |
| Human-*IFIT3* (R) | 5’-ACACCTTCGCCCTTTCATTTC-3’ |
| Human-*ISG15* (F) | 5’-CACCGTGTTCATGAATCTGC-3’ |
| Human-*ISG15* (R) | 5’-CTTTATTTCCGGCCCTTGAT-3’ |
| Human-*CXCL10* (F) | 5’-GGTGAGAAGAGATGTCTGAATCC-3’ |
| Human-*CXCL10* (R) | 5’-GTCCATCCTTGGAAGCACTGCA-3’ |
| Human-*OAS1* (F) | 5’-GACGATGAGACCGACGATCC-3’ |
| Human-*OAS1* (R) | 5’-CAGTCCTCTTCTGCCTGTGG-3’ |
| Human-*MX1* (F) | 5’-CAACCTGTGCAGCCAGTATGA-3’ |
| Human-*MX1* (R) | 5’-AGCCCGCAGGGAGTCAAT-3’ |
| Human-*GAPDH* (F) | 5’-GAGTCAACGGATTTGGTCGT-3’ |
| Human-*GAPDH* (R) | 5’-GACAAGCTTCCCGTTCTCAG-3’ |
| Human-*AUP1* (F) | 5’-GCGTCCTTCGCAGATTCGTA-3’ |
| Human-*AUP1* (R) | 5’-GGACCCTGACACTGTGATCC-3’ |
| Human-*UBE2G2* (F) | 5’-CCCAGAAGACACCTGCTTTGAG-3’ |
| Human-*UBE2G2* (R) | 5’-GGAGGATGGAAATGCAGACTCTC-3’ |
| Human-*XBP1s* (F) | 5’-CTGAGTCCGCAGCAGGTG-3’ |
| Human- *XBP1s* (R) | 5’-TCCAAGTTGTCCAGAATGCC-3’ |
| Human-*CPT1A* (F) | 5’-ACAGTCGGTGAGGCCTCTTA-3’ |
| Human-*CPT1A* (R) | 5’-CCACCAGTCGCTCACGTAAT-3’ |
| Human-*MGLL* (F) | 5’-GATGAGGGAACAGCCCGATT-3’ |
| Human-*MGLL* (R) | 5’-GGGTCTTCAGGTCCTGTTTCC-3’ |
| Human-*PRKAA2* (F) | 5’-TCGGCAAAGTGAAGATTGGAGA-3’ |
| Human-*PRKAA2* (R) | 5’-TCCAACAACATCTAAACTGCGA-3’ |
| Human-*STARD5* (F) | 5’-GAAGATTTGCCGGGAAGGCA-3’ |
| Human-*STARD5* (R) | 5’-TTCTCCTCGGTACAGGTTCCC-3’ |
| Human-*SOAT1* (F) | 5’-CGCGGGCCTCAGACAATAC-3’ |
| Human-*SOAT1* (R) | 5’-AGGGACTCCTTTGCAGGGTT-3’ |
| Human-*PPAR-γ* (F) | 5’-AGCAAACCCCTATTCCATGCT-3’ |
| Human-*PPAR-γ* (R) | 5’-TGTGTCAACCATGGTCATTTCTTG-3’ |
| Human-*SREBF1* (F) | 5’-ACTTCTGGAGGCATCGCAAGCA-3’ |
| Human-*SREBF1* (R) | 5’-AGGTTCCAGAGGAGGCTACAAG-3’ |
| Human-*FASN* (F) | 5’-GTCTTGAACTCCTTGGCGGA-3’ |
| Human-*FASN* (R) | 5’-AGGAAGATAGCCATGCCGAG-3’ |
| Human-*HMGCR* (F) | 5’-AGTGAGATCTGGAGGATCCAAG-3’ |
| Human-*HMGCR* (R) | 5’-CCCCACTATGACTTCCCAGG-3’ |
| Human-*ATGL* (F) | 5’-CCTGAGACGCCTCCATTACC-3’ |
| Human-*ATGL* (R) | 5’-GAAACCTCCCCTCTCAGTGC-3’ |
| Mouse-*Ifnb1* (F) | 5’-CTCCAGCTCCAAGAAAGGAC-3’ |
| Mouse-*Ifnb1* (R) | 5’-TGGCAAAGGCAGTGTAACTC-3’ |
| Mouse-*Ifit1* (F) | 5’-CTGAGATGTCACTTCACATGGAA-3’ |
| Mouse-*Ifit1* (R) | 5’-GTGCATCCCCAATGGGTTCT-3’ |
| Mouse-*Isg15* (F) | 5’-AGAAGCAGATTGCCCAGAAG-3’ |
| Mouse-*Isg15* (R) | 5’-TGCGTCAGAAAGACCTCATAGA-3’ |
| Mouse-*Cxcl10* (F) | 5’-ATCATCCCTGCGAGCCTATCCT-3’ |
| Mouse-*Cxcl10* (R) | 5’-GACCTTTTTTGGCTAAACGCTTTC-3’ |
| Mouse-*Oas1* (F) | 5’-ATCTGCATCAGGAGGTGGAG-3’ |
| Mouse-*Oas1* (R) | 5’-GCTCCGTGAAGCAGGTAGAG-3’ |
| Mouse-*Gapdh* (F) | 5’-GTCATCCCAGAGCTGAACG-3’ |
| Mouse-*Gapdh* (R) | 5’-TCATACTTGGCAGGTTTCTCC-3’ |
| Mouse-*Aup1* (F) | 5’-TCTCGCTCACAGAGTCAAGGAG-3’ |
| Mouse-*Aup1* (R) | 5’-CGAGCAGGTTTGTGATGGTCAAG-3’ |
| Mouse-*Ube2g2* (F) | 5’-GCCATCCTGAGTTTCCCACT-3’ |
| Mouse-*Ube2g2* (R) | 5’-CTGCCATCAGGATAGATGTTGGG-3’ |
| HSV-1 (F) | 5’-CATCACCGACCCGGAGAGGGAC-3’ |
| HSV-1 (R) | 5’-GGGCCAGGCGCTTGTTGGTGTA-3’ |
| VSV-1 (F) | 5’-ACGGCGTACTTCCAGATGG-3’ |
| VSV-1 (R) | 5’-CTCGGTTCAAGATCCAGGT-3’ |
| VACV (F) | 5’-TCAGAATCTAATGATGACGT-3’ |
| VACV (R) | 5’-GCCTAGTAACTCTCCTACAT-3’ |

**Supplementary Table** **2:** **The sequences of short hairpin RNAs and single guide RNAs**

| Primer designation | Primer sequence |
| --- | --- |
| sg-*AUP1* | 5’-GGACCATGTGTGCGGTGCTA-3’ |
| sh-*AUP1* | 5’-GCAGAGCACATGAAGCGACAA-3’ |
| sh-*UBE2G2* | 5’-GAGATTTACCTGTGAGATGTT-3’ |
| sh-*STING*-1 | 5’-GCATGGTCATATTACATCGGA-3’ |
| sh-*STING*-2 | 5’-GCCCGGATTCGAACTTACAAT-3’ |
| sh-*Aup1* | 5’-CGTTTCAGTTCGTGGCCATTT-3’ |
| sh-*Ube2g2*-1 | 5’-GCAGTTCTACAAGATCGCCAA-3’ |
| sh-*Ube2g2*-2 | 5’-CCTGAGTTTCCCACTTGACTA-3’ |
